# Comprehensive genetic screening of early-onset dementia patients in an Austrian cohort-suggesting new disease-contributing genes

**DOI:** 10.1186/s40246-023-00499-z

**Published:** 2023-06-17

**Authors:** Sara Silvaieh, Theresa König, Raphael Wurm, Tandis Parvizi, Evelyn Berger-Sieczkowski, Stella Goeschl, Christoph Hotzy, Matias Wagner, Riccardo Berutti, Esther Sammler, Elisabeth Stögmann, Alexander Zimprich

**Affiliations:** 1 Department of Neurology, Medical University of Vienna, Vienna, Austria; 2 Comprehensive Center for Clinical Neurosciences and Mental Health, Medical University of Vienna, Vienna, Austria; 3 Institute of Human Genetics, School of Medicine, Technical University of Munich, Munich, Germany; 4 Institute of Neurogenomics, Helmholtz Centrum, Munich, Germany; 5 Molecular and Clinical Medicine, Ninewells Hospital and Medical School, University of Dundee, Dundee, DD1 9SY UK; 6 Medical Research Council Protein Phosphorylation and Ubiquitylation Unit, School of Life Sciences, University of Dundee, Dundee, DD1 5EH UK

**Keywords:** Early-onset dementia, Whole-exome sequencing, Genetic variants

## Abstract

Early-onset dementia (EOD), with symptom onset before age 65, has a strong genetic burden. Due to genetic and clinical overlaps between different types of dementia, whole-exome sequencing (WES) has emerged as an appropriate screening method for diagnostic testing and novel gene-finding approaches. We performed WES and C9orf72 repeat testing in 60 well-defined Austrian EOD patients. Seven patients (12%) carried likely disease-causing variants in monogenic genes, PSEN1, MAPT, APP, and GRN. Five patients (8%) were APOE4 homozygote carriers. Definite and possible risk variants were detected in the genes TREM2, SORL1, ABCA7 and TBK1. In an explorative approach, we cross-checked rare gene variants in our cohort with a curated neurodegeneration candidate gene list and identified DCTN1, MAPK8IP3, LRRK2, VPS13C and BACE1 as promising candidate genes. Conclusively, 12 cases (20%) carried variants relevant to patient counseling, comparable to previously reported studies, and can thus be considered genetically resolved. Reduced penetrance, oligogenic inheritance and not yet identified high-risk genes might explain the high number of unresolved cases. To address this issue, we provide complete genetic and phenotypic information (uploaded to the European Genome-phenome Archive), enabling other researchers to cross-check variants. Thereby, we hope to increase the chance of independently finding the same gene/variant-hit in other well-defined EOD patient cohorts, thus confirming new genetic risk variants or variant combinations.

**Supplementary Information:**

The online version contains supplementary material available at 10.1186/s40246-023-00499-z.

## Background

Early-onset dementia (EOD), with symptoms appearing before the age of 65, such as early-onset Alzheimer’s disease (EOAD) and frontotemporal dementia (FTD), represents about 1–2% of all dementia cases [1]. EODs are typically thought of as disease forms with highly penetrant genetic contributions [2–4]. The disease commonly aggregates within families in these patients, with 10–15% showing an autosomal dominant pattern of inheritance [5]. Nonetheless, although a high heritability is known, a monogenetic cause or clear genetic risk factor is only identified in a small part of these cases. In recent studies of EOD cases, whole-exome sequencing (WES) yielded an overall diagnostic rate of ~ 16–30% [6–9]. Nevertheless, clinical genetic testing is not routinely performed for EOD, and WES remains poorly studied as a potential genetic diagnostic tool for late-onset neurodegenerative disorders. In this study, we systematically assessed the genetic background of EOD by comprehensive genetic screening of 60 clinically well-characterized patients using WES, copy number variation (CNV) analysis and C9orf72 repeat-primed PCR-analysis.

Our objectives were as follows: (i) to evaluate the genetic burden in the patients caused by mutations in monogenic disease genes and established risk factors, (ii) to suggest possible new candidate genes for EOD, (iii) and to share detailed genotypic and phenotypic data with the scientific community in a publicly accessible database.

## Results

Genetic findings are presented according to the implication of the variant on an individual basis. In this context, we structured our patient cohort into three categories: (i) causal variants and variants that contribute to a large extent to the disease and whose carriers are therefore considered to be resolved from a genetic-diagnostic perspective (carriers of variants relevant for diagnostics), (ii) variants in established risk genes that confer medium to low risk for an individual to develop dementia (carriers of well-established and potential new risk variants) and (iii) potential disease-contributing variants in non-classical dementia genes (possible new candidate genes for dementia). Essential clinical and genetic characteristics of all 60 patients are shown in Table 1. A detailed clinical description of each patient is given in Additional file 1.

**Table 1:** Basic clinical and genetic characteristics of all 60 EOD patients

ID	Diagnosis	AAO (y)	Sex	FH	APOE	Gene	Variant	Position	Transcript	CADD	ClinVar	Significance for disease
**EOD-1**	**AD**	**54**	**f**	**3**	**E2/E3**	***PSEN1***	**c.356C>T; p.T119I**	**chr14:73640291-73640291**	**NM_000021.3**	**24.4**	**LP**	**Relevant for diagnosis**
**EOD-2**	**bvFTD**	**44**	**f**	**1**	**E4/E3**	***MAPT***	**c.1907C>T; p.P636L**	**chr17:44087755-44087755**	**NM_001123066.3**	**34.0**	**P**	**Relevant for diagnosis**
						*TREM2*	c.184G>A; p.R62C	chr6:41129208-41129208	NM_001271821.1	25.5	n.r.	Risk modifier
						*APOE*						Risk modifier
**EOD-3**	AD	45	f	2	E3/E3							
**EOD-4**	AD	51	f	4	E4/E3	*APOE*						Risk modifier
**EOD-5**	nfPPA	58	f	2	E3/E2							
**EOD-6**	AD	56	f	3	E3/E3							
**EOD-7**	AD/PCA	56	f	4	E3/E3							
**EOD-8**	bvFTD	56	m	4	E3/E3	*BACE1*	c.1427T>C; p.M476T	chr11:117160361-117160361	NM_012104.3	26.4	n.r.	Unknown
						*VPS13C*	c.9757A>G; p.S3253G	chr15:62173781-62173781	NM_020821.2	29.5	n.r.	Unknown
**EOD-9**	AD	55	f	3,5	E4/E3	*APOE*						Risk modifier
**EOD-10**	AD	58	f	3,5	E3/E3							
**EOD-11**	AD	63	m	4	E3/E3							
**EOD-12**	mixed dementia (AD+VD)	55	m	3,5	E4/E3	*APOE*						Risk modifier
**EOD-13**	AD	61	m	4,5	E3/E3							
**EOD-14**	AD/lpPPA	61	m	4	E4/E3	*APOE*						Risk modifier
						*VPS13C*	c.4300C>T; p.V1434I	chr15:62244179-62244179	NM_020821.2	24.8	n.r.	Unknown
**EOD-15**	nfPPA	64	m	2	E3/E3	*DCTN1*	c.2218C>T; p.E740K	chr2:74594514-74594514	NM_004082.4	24.0	n.r.	Unknown
**EOD-16**	AD	56	f	4	E3/E3							
**EOD-17**	AD (PD)	60	m	1	E4/E3	*APOE*						Risk modifier
						*MAPK8IP3*	g.chr16:1816528 A>G; c.2817-2A>G	chr16:1816528-1816528	NM_015133.3	22.3	n.r.	Unknown
**EOD-18**^a^	**AD**	**47**	**m**	**4**	**E3/E3**	***APP***	**g.chr.21:(?_26958019)-(27852747_?)dup**				**P**	**Relevant for diagnosis**
						*ABCA7*	c.2914C>T; p.P972S	chr19:1051537-1051537	NM_019112.3	25.3	n.r.	Potential risk modifier
**EOD-19**	**AD**	**51**	**m**	**1**	**E3/E3**	***APP***	**g.chr.21:(?_27253981)-(27542937_?)dup**				**P**	**Relevant for diagnosis**
**EOD-19 (2)**^b^	**AD**	**47**	**m**	**1**	**E3/E3**	***APP***	**g.chr.21:(?_27253981)-(27542937_?)dup**				**P**	**Relevant for diagnosis**
**EOD-20**	AD	57	m	4,5	E3/E3	*LRRK2*	c.7397T>A; p.L2466H	chr12:40760814-40760814	NM_198578.3	25.7	VUS	Unknown
**EOD-21**	**CAA**	**54**	**m**	**3,5**	**E4/E4**	***APOE***						**Relevant for diagnosis**
**EOD-22**	**AD**	**49**	**m**	**4**	**E4/E4**	***APOE***						**Relevant for diagnosis**
**EOD-23**	**AD**	**36**	**f**	**1**	**E3/E3**	***PSEN1***	**c.617G>A; p.G206D**	**chr14:73659420-73659420**	**NM_000021.3**	**31.0**	**P**	**Relevant for diagnosis**
**EOD-24**	**AD**	**53**	**m**	**3,5**	**E4/E4**	***APOE***						**Relevant for diagnosis**
**EOD-25**	**AD**	**51**	**f**	**3,5**	**E4/E4**	***APOE***						**Relevant for diagnosis**
**EOD-26**	AD	56	f	4	E3/E3	*DCTN1*	c.2980G>C; p.P994A	chr2:74590268-74590268	NM_023019.3	17.3	VUS	Unknown
						*MAPK8IP3*	c.2087G>A; p.R696H	chr16:1814180-1814180	NM_015133.3	31.0	n.r.	Unknown
**EOD-27**	AD	57	f	4	E4/E3	*APOE*						Risk modifier
**EOD-28**	AD	54	m	4	E3/E3							
**EOD-29**	AD	54	m	4	E3/E3							
**EOD-30**	AD	64	m	4	E3/E3							
**EOD-31**	mixed dementia (AD+VD)	58	m	3,5	E3/E3							
**EOD-32**	FTD/svPPA	61	m	4	E3/E3							
**EOD-33**	AD	62	f	4,5	E4/E3	*APOE*						Risk modifier
						*DCTN1*	c.521G>A; p.S174L	chr2:74598788-74598788	NM_004082.4	24.4	VUS	Unknown
**EOD-34**	AD	59	f	2	E4/E3	*APOE*						Risk modifier
**EOD-35**	AD	55	m	3,5	E4/E3	*APOE*						Risk modifier
**EOD-36**^c^	AD	64	m	2	E4/E3	*TREM2*	c.140G>A; p.R47H	chr6:41129252-41129252	NM_018965.3	9.7	LB	Risk modifier
						*APOE*						Risk modifier
**EOD-37**	AD	52	f	3,5	E3/E3	*LRRK2*	c.7397T>A; p.L2466H	chr12:40760814-40760814	NM_198578.3	25.7	VUS	Unknown
**EOD-38**	AD	52	f	3,5	E4/E3	*APOE*						Risk modifier
**EOD-39**	AD	63	f	3	E4/E3	*APOE*						Risk modifier
**EOD-40**	AD	55	f	4	E4/E3	*APOE*						Risk modifier
**EOD-41**	AD	58	m	3,5	E3/E3							
**EOD-42**	AD	39	m	4	E3/E2							
**EOD-43**	AD	63	m	4	E3/E3	*VPS13C*	c.3148A>G; p.I1050V	chr15:62256964-62256964	NM_020821.2	0.001	VUS	Unknown
**EOD-44**	AD/lpPPA	58	f	3,5	E3/E3	*SORL1*	c.3014T>G; p.M1005R	chr11:121430331-121430331	NM_003105.5	27.9	n.r.	Potential risk modifier
**EOD-45**	AD	65	m	4	E3/E3							
**EOD-46**	CBS+ AD	51	f	3,5	E3/E3	*SORL1*	c.4606G>A; p.G1536S	chr11:121474988-121474988	NM_003105.5	25.2	B	Risk modifier
**EOD-47**	AD	54	f	4	E3/E3							
**EOD-48**	bvFTD	57	m	4	E3/E3							
**EOD-49**	FTD/nfPPA+ALS	58	m	4	E3/E3	*TBK1*	c.986T>C; p.L276P	chr12:64875636-64875636	NM_013254.3		n.r.	Potential risk modifier
						*VPS13C*	c.7436T>C; p.I2429T	chr15:62212307-62212307	NM_020821.2		n.r.	Unknown
**EOD-50**	**FTD (bvFTD + nfPPA)**	**55**	**f**	**3,5**	**E4/E3**	***PGRN***	c.328C>T; p.R110*	**chr17:42427098-42427098**	**NM_002087.3**	**29.4**	**P**	**Relevant for diagnosis**
						*APOE*						Risk modifier
**EOD-51**	FTD/svPPA	62	f	4	E3/E3							
**EOD-52**	AD	57	m	4	E4/E3	*APOE*						Risk modifier
**EOD-53**	**AD**	**57**	**m**	**4**	**E4/E4**	***APOE***						**Relevant for diagnosis**
						*LRRK2*	c.7377G>A; p.M2459I	chr12:40758839-40758839	NM_198578.3	17.7	n.r.	Unknown
**EOD-54**	AD	59	m	1	E4/E3	*APOE*						Risk modifier
**EOD-55**	AD	49	m	4	E3/E3							
**EOD-56**	AD	61	m	3,5	E3/E3							
**EOD-57**	AD/lpPPA	57	f	4	E3/E3							
**EOD-58**	AD +VD	64	f	3	E3/E3	*DCTN1*	c.823C>T; p.R141C	chr2:74598126-74598126	NM_004082.3	29.3	VUS	Unknown
**EOD-59**	bvFTD	52	m	4	E4/E3	*APOE*						Risk modifier
**EOD-60**	**AD**	**49**	**f**	**3**	**E3/E3**	***APP***	**c.2092G>A; p.V586I**	**chr21:27264096 **	**NM_201413.3**	**28.2**	**P**	**Relevant for diagnosis**

^a^EOD-18: The APP duplication of was confirmed to be ‘de novo’. Both parents did not show this duplication

^b^EOD-19 (2) is the brother of EOD19. He was also affected by AD and carrier of the same duplication. EOD 19 (2) was not included in the analyses of AAO and FH

^c^EOD-36: ClinVar assessment of TREM2 p.R47H of LB (likely benign) refers to Nasu-Hakola disease. However, p.R47H is an established risk variant for dementia [15]

### Carriers of variants relevant for diagnostics

We defined carriers of variants relevant for diagnostics as those carrying mutations that explain the onset of disease to a large extent. These variants confer high age-dependent penetrance. Patients and family members carrying these variants should be offered genetic counseling. This group includes carriers of pathogenic and likely pathogenic variants in autosomal dominant AD genes as defined by the American College of Medical Genetics and Genomics and the Association for Molecular Pathology (ACMG) [10]. We also included individuals homozygous for the APOE4 allele in this category. Although this genotype is not fully penetrant and cannot be classified as causal for EOD, the lifetime risk of carriers for developing cognitive deficits at age 85 is estimated to be approximately 80% [11].

In total, we identified 12 patients (20%) in this group, seven carrying pathogenic variants in the autosomal dominant genes PSEN1 (*n* = 2), MAPT (*n* = 1), APP (*n* = 3) and PGRN (*n* = 1) and five homozygous APOE4 allele carriers (highlighted in bold in Table 1).

Notably, testing the C9orf72 repeat length revealed no pathological repeat expansion in our cohort.

The identified mutations in these pathogenic and likely pathogenic variants correspond with the clinical diagnosis.

### Carriers of well-established and potential new risk variants

We defined carriers of well-established risk variants as those harboring variants conferring low to medium risk for dementia, i.e., variants that increase risk but are not causal for disease. Overall, 33% of our study cohort were carriers of established risk variants, which includes APOE4 heterozygote- and TREM2 risk variant carriers (*n* = 20). Eighteen patients were heterozygote carriers of the APOE4 allele. Two patients (EOD-2 and EOD-36) carried the well-established TREM2 risk variants p.(R47H) and p.(R62C), respectively, [12, 13].

We defined potential new risk variants as rare variants with unknown significance in established risk genes. We identified three patients carrying missense variants in two genes, *ABCA7* [p.(P972S) in EOD-18] and SORL1 [p.(M1005R) in EOD-44 and p.(G1536) in EOD-46]. Both genes have been widely described as implicated in late- and early-onset forms of AD [14, 15]. However, none of the three variants has yet been reported in association with dementia. SORL1-p.(G1536S) is reported to be very frequent in the Ashkenazi Jewish population, with 1 out of 130 individuals carrying this variant in the heterozygous state as indicated in the gnomAD database (allele count: 40/10370; MAF:0.0038). Interestingly, located only one amino acid next to G1536S, another variant, p.(G1535N), has previously been described to cause a maturation defect of the protein, leading to reduced expression and excretion deficiency [16]. Both *ABCA7*-p.(P972S) and *SORL1*-p.(M1005R) have not been reported in the gnomAD yet.

### Possible new candidate genes for dementia

Next, in a more exploratory approach, we investigated whether non-classical dementia genes might contribute to disease risk. We compiled a list of 564 genes described in the literature as linked to different forms of neurodegeneration. This list included, for example, suggested but unproven candidate genes in various neurodegenerative diseases, low-risk genes from genome-wide association studies, or candidate genes from animal or functional studies (for the complete list of genes, see Additional file 1: Table S2). Those genes were then cross-checked with all rare, predicted protein-altering variants (MAF < 0.01%) present in our patients. We subsequently selected candidate genes based on one of the following criteria: (i) repeated occurrence of the same variant in different patients, (ii) occurrence of different variants in the same gene in different patients or (iii) variants with a strong association with pathways involved in dementia pathogenesis. Here, we identified hits in five genes (DCTN1, JIP3 aka MAPK8IP3, LRRK2, BACE1 and VPS13C).

The patients' genetic and clinical information are abbreviated as follows: sex (male/female); diagnosis (Dg); age at onset (AAO); family history (FH); interpretation of the variant according to ACMG (ClinVar); genotype counts in the Genome Aggregation Database (gnomAD).

Whenever possible, we carried out genetic tests on symptomatic relatives of patients. Unfortunately, there were no more symptomatic relatives who were alive (e.g., EOD-17, EOD-37), or the patients were not in contact with them (e.g., EOD-15). For ethical reasons, we did not perform genetic testing on the (asymptomatic) offspring unless the asymptomatic offspring specifically requested it.

#### DCTN1

We identified four patients with rare missense variants in the dynactin subunit 1 gene (DCTN1). (i) EOD-15: p.(E740K); male; Dg: AD; AAO: 64; FH: positive (sister and cousin: AD); gnomAD: 1/251.410. (ii) EOD-26: p.(P994A); female; Dg: AD; AAO:56; FH: negative; ClinVar: VUS in a case of distal motor neuronopathy; gnomAD: not present. (iii) EOD-33: p.(S174L); female, Dg: AD; AAO:42; FH: unknown; ClinVar: VUS in a patient with no disclosed clinical details; gnomAD: 1/221.382 (iv) EOD-58: p.(R141C); female; Dg: mixed dementia (AD + VD); AAO: 57; FH: negative; ClinVar: VUS; gnomAD: not present.

Autosomal dominant mutations in the DCTN1 gene cause Perry syndrome, characterized by Parkinsonism, psychiatric symptoms, weight loss and central hypotension (OMIM#168605) [17, 18]. However, patients with pathogenic DCTN1 mutations may exhibit more diverse phenotypes, such as progressive supranuclear palsy- and/or FTD-like syndromes as well as distal hereditary motor and sensor neuropathies [18]. DCTN1, which encodes p150glued, is the largest subunit of the dynactin complex and is involved in microtubule binding and molecular transport [19]. An interesting link to monogenic dementia has recently been revealed, as DCTN1 has been found in a yeast two-hybrid screen to directly interact with APP and tau (encoded by MAPT), suggesting that these three proteins are linked at a molecular level [20]. Also supportive of the role of DCTN1 in EOD is that three of four of our variants are listed in clinico-genetic databases. p.(S174L), p.(P994A) and p.(R141C) in ClinVar, (VUS) and p.(S174L), listed in the in-house database of the Institute of Human Genetics in Munich. Here, two out of 25.000 individuals (one with hereditary motor and sensor neuropathy and one with ALS) were found to carry the p.(S174L) variant. All pathogenic mutations described so far for Perry syndrome cluster in exon 2, within the CAP-Gly domain. The variants identified in our patients are all located outside this domain, in exon 8, p.(R141C); 9, p.(S174L); 19, p.(E740K) and 28, p.(P994A), respectively, and thus cannot be considered as pathogenic for Perry disease. However, there have also been reports of DCTN1-variants linked to FTD and ALS variants outside the CAP-Gly domain [18].

#### JIP3; MAPK8IP3

We identified two patients with rare missense variants in the C-jun-amino-terminal kinase interacting protein 3 (JIP3), aka mitogen-activated protein kinase 8 interacting protein 3 (MAPK8IP3) gene. MAPK8IP3 is an adaptor protein of the kinesin-1 complex and is essential for axonal transport in neurons [21–23]. (i) EOD-17: a direct splice AG > GG variation in intron 22 adjacent to exon 23 (GRCh37/hg19: g.chr16:1816528 A > G; c. 2817-2A > G, NM_015133); male; Dg: AD + PD; AAO: 60; FH: positive (mother, maternal aunts, grandmother: unspecified dementia; cousin: EOAD); gnomAD; not present. Transcriptome analysis of the patients’ blood RNA revealed aberrant splicing between exons 22 and 23, which indicates that this variant causes a loss of function (LoF) (Additional file 1). (ii) EOD-26: p.(R696H); female; AD; AAO: 56; FH: negative; gnomAD: not present. Notably, the same patient also carried the DCTN1-p.(994) variant (see above). De novo missense mutations clustering in specific protein regions were recently identified in patients with childhood-onset developmental delay and epilepsy [24, 25]. Notably, according to gnomAD, eleven out of > 140.000 individuals are predicted to carry heterozygous LoF variants in MAPK8IP3, indicating that pathogenic variants associated with developmental delay are not caused by LoF but rather gain of function variants. In line with these considerations, our patients showed no signs of developmental delay or structural brain abnormalities.

Recent observations suggest a role of MAPK8IP3 in neurodegeneration. Mice lacking Mapk8ip3 show an accumulation of lysosomal vesicles containing amyloid processing enzymes and increased production of toxic species of amyloid beta (Aβ) [26]. Furthermore, most indicative of a potential role of MAPK8IP3 in dementia is the significant association of rare LoF variants with the disease, as revealed by a large-scale WES study including ~ 7.000 AD patients [27]. It is, therefore, tempting to speculate that reduced protein levels confer risk in neurodegeneration.

#### LRRK2

Leucine-rich repeat kinase 2 (LRRK2) is a major causative gene of late-onset familial Parkinson's disease (PD) (OMIM#607060). Remarkably, some of the clearly PD associated mutations have also been found in patients suffering from atypical PD and tauopathies [28–33]. Three patients in our cohort were identified to carry rare variants in the LRRK2 gene. Two patients carry the same LRRK2 variant. (i) EOD-20: p.(L2466H); male; Dg: AD; AAO: 57; FH: unknown; (ii) EOD-37: p.(L2466H); female; Dg: AD, AAO: 52; FH: positive (mother, unspecified dementia); gnomAD: 1/250,300. Although both patients originate from Serbia, they do not seem closely related as the number of shared SNPs is not higher than in any other pair of unrelated individuals (< 7% of rare variants with MAF < 0.01%). p.(L2466H) has been identified in previous PD case–control studies, but no clear association with PD has been established [34]. (iii) EOD-53: p.(M2459I); male; Dg: AD; AAO: 57; FH: negative; gnomAD: not present. Recent work has revealed that LRRK2 phosphorylates a subgroup of 14 endogenous RabGTPases. [35, 36]. Consistent with Rab proteins comprising disease-relevant substrates, all established high penetrant PD-causing mutations enhance LRRK2-mediated Rab protein phosphorylation and measuring Rab10 phosphorylation has become a reliable readout for pathogenic LRRK2 mutations[37]. To address the question of whether p.(L2466H) increases LRRK2 kinase activity, we assessed Rab10 phosphorylation in neutrophils and monocytes derived from peripheral blood of the male patient (EOD-20) carrying the p.(L2466H) variant and his asymptomatic mother, who also carries this variant, using quantitative multiplexed immunoblot analysis, as recently described [38, 39]. In addition, we employed a cellular HEK293 overexpression system of LRRK2 variants to directly assess the impact of the LRRK2 L2466H variant on endogenous Rab10 phosphorylation at Threonine 73 in comparison to LRRK2 wildtype and LRRK2 R1441G as a kinase-activating controls as previously described [37]. LRRK2 dependent Rab10 phosphorylation was neither observed in patient derived peripheral blood neutrophils nor in the cellular HEK293 assay (Additional file 1). These results are in keeping with a recent large-scale overexpression study, where 100 LRRK2 variants were tested, including L2466H [37]. These functional results and the fact that the LRRK2 p.(L2446H) lacks genetic association with PD, make it unlikely that p.(L2466H) promotes disease in the same way as typical high susceptibility PD variants, i.e., increasing Rab phosphorylation. Notably, the fact that the non-affected mother, who died at the age of 83, also carries the variant indicates that p.(L2466H) cannot be fully penetrant. Both p.(L2466H) and p.(2459I) are located in the C-Terminal part of the protein within the WD40 domain. Taken together, our data do not support a clear pathogenic effect of p.(L2466H), at least concerning kinase function. However, it would be interesting to investigate whether p.(L2466H) or p.(M2459I) might exert a pathogenic effect via a non-kinase-activating mechanism.

#### BACE1

One patient was found to carry a novel variant in the β-site APP-cleaving enzyme 1 (BACE1) gene. (i) EOD-8: p.(M476T); male; Dg: bvFTD; AAO: 56; FH: negative; gnomAD: not present. BACE1 is a key enzyme in the formation of the Aβpeptide. It cleaves APP to generate a C-terminal fragment (β-CTF). The β-CTF fragment is then further processed by the γ-secretase to produce Aβ fragments.

Interestingly, especially since the variant is located in the transmembrane region, p.(M476T) replaces a hydrophobic amino acid (methionine) with a polar amino acid with an uncharged side chain (threonine). The transmembrane domain is necessary for the access of BACE1 enzymatic activity to the cellular APP substrate [40]. Although mutations in the APP gene near the β-secretase sites can cause autosomal dominant AD [41–43], to date, no gene variations in the BACE1 gene have been directly linked to the disease. However, in a recent study, epigenetic changes in the BACE1 gene were associated with AD. A highly significant hypomethylation of the BACE1 enhancer region in prefrontal cortex neurons of AD patients was linked with increased expression of BACE1. Intriguingly, these changes occurred early in the disease process and can be assumed to have a major impact on pathogenesis [44]. This suggests that genetic determinants, possibly via epigenetic factors, might also increase the risk for AD.

#### VPS13C

We identified four patients with rare variants in the vacuolar protein sorting-associated protein 13C (VPS13C). (i) EOD-14: p.(V1434I); male; Dg: AD/ logopenic variant primary progressive aphasia (IpPPA); AAO:61; FH: negative; gnomAD: not present. (ii) EOD-43: p.(I1050V); male; Dg: AD; AAO:63; FH: negative; gnomAD: 22/281516. (iii) EOD-8: p.(S3253G); male; Dg: bvFTD; AAO: 56; FH: negative; gnomAD: 5/281508. This patient also carried the BACE1- p.(M476T) variant. (iv) EOD-49: p.(I2429T); male; Dg: FTD/nfPPA; AAO:58; FH: negative; gnomAD: not present. VPS13C is involved in the regulation of mitochondrial function and the regulation of PINK1/PRKN-mediated mitophagy. Bi-allelic LoF variants in the VPS13C gene cause autosomal recessive, early-onset PD. Several indications point to the role of VPS13C in dementia. Bi-allelic VPS13C PD patients show a strong association with early cognitive decline [45]. Furthermore, rare heterozygous VPS13C variants were recently found to be associated with DLB [46]. We performed a survey for associations of rare VPS13C variants with disease phenotypes using UK biobank data, including 50.000 individuals [47]. Interestingly, the strongest association was found with dementia (*p* < 2 × 10–3) (https://ukb-50kexome.leelabsg.org/gene/VPS13C); However, it has to be mentioned that only 60 cases with dementia were included in this calculation.

### Age at disease onset (AAO)

Next, we investigated whether the groups defined in this study showed differences in their AAO.

**Fig. 1:**
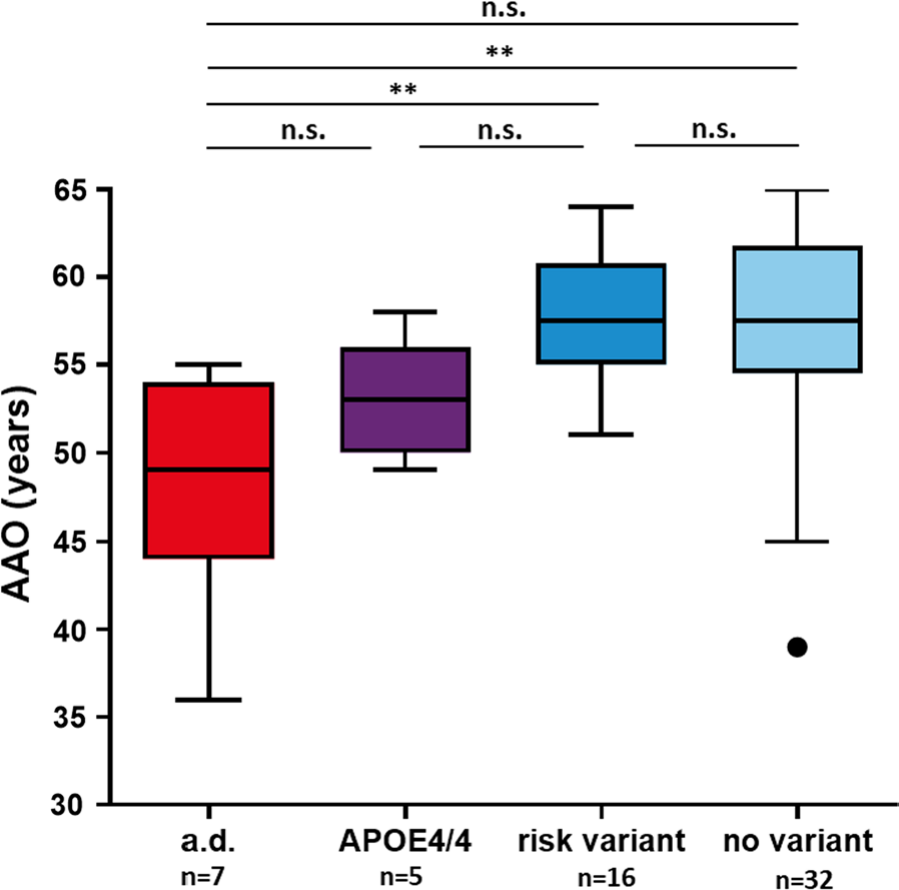
The boxplot compares the AAO in patients carrying autosomal dominant variants (a.d., *n* = 7), APOE4/4 carriers (*n* = 5), established risk variants (*n* = 16) and those without established variants (*n* = 32). Significantly earlier onset was found in the a.d. group compared to the risk variant (*p* = 0.003) and no variant (*p* = 0.002) group. Note that the difference to APOE4 homozygote carriers was statistically not significant (*p* = 0.385). Significance was lost when correcting for multiple testing comparing APOE4/4 carriers to patients carrying risk variants (*p* = 0.036, adj. *p* = 0.138) and to patients with no variant (*p* = 0.048, adj.*p* = 0.138) No difference was found between carriers of risk variants) compared to the rest of the cohort (*p* = 0.849). Statistical analysis was performed using Mann–Whitney U test and corrected for multiple testing using the Holm-Sidak method; boxplots represent median, quartiles and outliers according to the Tukey method; a.d. = autosomal dominant variants in PSEN1, MAPT, APP, GRN; APOE4/4 = APOE4 homozygote carriers; risk variant = patients with established risk variants, including TREM2 p.(R417H) and APOE4 heterozygotes; no variant = patients with no established risk variants nor variants in established risk genes (including patients with potential new risk variants and new candidate gene variants for dementia). Note that one patient, EOD-2, who carries two established risk variants (heterozygous APOE4 and TREM2 p.(R62C) and one autosomal dominant variant MAPT p.(P636L), was only assigned to the group of autosomal dominant carriers

We found a highly significant earlier AAO in “Carriers of variants relevant for diagnostics” section (*n* = 12, median AAO = 51, IQR = 48–54) compared to the rest of the cohort (*n* = 48, median AAO = 58, IQR = 55–61; *p* < 0.0001). There was also a non- significant tendency towards an earlier AAO in carriers of autosomal dominant variants PSEN1, APP, PGRN, and MAPT (*n* = 7, median AAO = 49, IQR = 44–54) compared to APOE4 homozygous patients (*n* = 5, median AAO = 53, IQR = 50–56), (*p* = 0.39, Fig. 1). The subgroup of APOE4 homozygote carriers showed significantly earlier AAO compared to carriers of established risk variants (APOE4/4: *n* = 5, median AAO = 53, IQR = 50–56 vs. risk variants: *n* = 16, median AAO = 58, IQR = 55–61, *p* = 0.036, adj. *p* = 0.138) and the rest of the cohort without established variants (no variant: *n* = 32, AAO = 58, IQR = 55–62; *p* = 0.048, adj. *p* = 0.138), though significance was lost after correcting for multiple comparisons.

Comparison of the AAO in carriers of established risk variants (including APOE4 heterozygote and TREM2-R47H carriers, *n* = 16) revealed no difference in their AAO to the rest of the cohort (*n* = 32; median AAO = 58 in both groups; *p* = 0.85). Notably, the patients also carrying variants relevant for diagnostics (*n* = 12) were excluded from the analysis of risk variants.

### Family history

Next, we were interested in whether the groups, as defined in this study, show differences in their family history of dementia. For this purpose, all patients were given a score between 1 and 4 as per Goldman et al. [5, 48], where 1 represents the strongest degree of a familial background of dementia and 4 the weakest. Patients with an unknown family history were rated as 4.5 (see “Materials and methods” section).

**Fig. 2:**
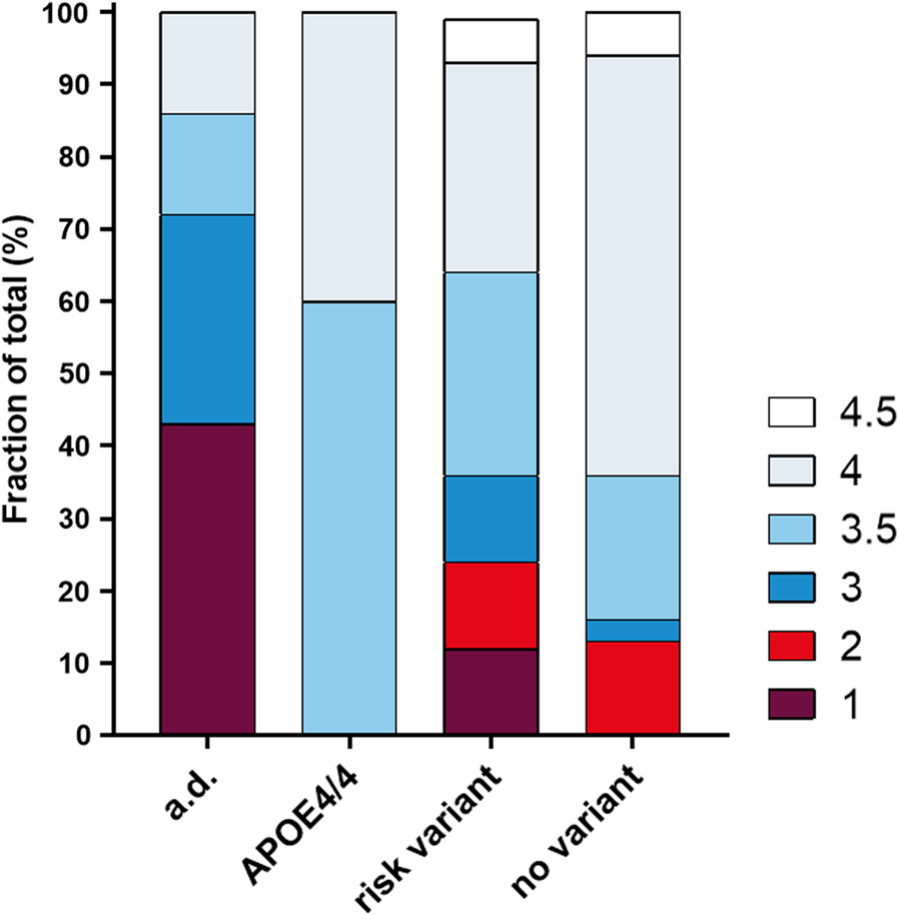
The bar graph shows the relative frequency of strong FH (Goldman score 1–2), moderate to low FH (3–3.5) and negative or unknown FH (4–4.5) in patients with autosomal dominant variants (a.d.; PSEN1, MAPT, APP duplication/missense mutation, GRN), APOE4/4: Homozygote APOE4 carriers; risk variant: well-established risk variants (TREM2, APOE4) and no variants in established risk genes or of unknown significance

Carriers of autosomal dominant mutations showed the highest degree of positive family history, with 43% (*n* = 3/7) having the highest score of 1, defined as the presence of at least three affected family members in two generations. Two patients scored 3, defined as one other affected family member with young-onset dementia within the family; of note, one patient within this group, EOD-18, carried an APP duplication, which had been confirmed to have occurred “de novo”. This patient had no family history of dementia. Thus, one should keep in mind that a negative family history does not automatically imply the absence of a highly penetrant mutation. Not unexpectedly, APOE4 homozygote carriers showed only moderate familial background of dementia. Three out of five patients scored 3.5, defined as one other affected family member with an age of onset above 65. This reflects the well-known semi-dominant inheritance mechanism of the APOE4 allele: first-degree relatives (parents and children) inherit only one APOE4 allele which, in its heterozygote state, is associated with only moderate disease risk (odds ratio ~ 3) and usually late disease onset (Fig. 2).

## Discussion

In this study, we conducted a comprehensive analysis of high and moderate genetic risk factors in an Austrian cohort of early-onset dementia (EOD).

For this, we performed extensive genetic screening using WES, copy number variation (CNV) analysis and C9orf72 repeat-primed PCR-analysis. We identified 12 patients (20%) with high penetrant risk variants whom we defined as “Carriers of variants relevant for diagnostics” section (these include pathogenic variants in APP, MAPT, PSEN1, GRN and the APOE4/4 genotype). In this group, we also included APOE4 homozygotes, although this genotype is not highly penetrant and therefore does not have the same diagnostic significance as causal variants in autosomal dominant genes. However, their lifetime risk for developing dementia or cognitive impairment is considerably high, with estimates ranging from 30 to 50% [49] and up to over 80% in 80-year-olds [11]. Additionally, amyloid deposition was shown to start earlier in life than in non-carriers and genotyping is recommended when considering recently approved anti-amyloid monoclonal antibodies [50]. For most of our patients (80%), we could not find highly penetrant mutations or the APOE4/4 genotype, thus leaving the overwhelming part of our patients “genetically unexplained”. This is in accordance with previous WES studies in EOD, where a diagnostic yield of only 16–30% has been observed [6–9]. Notably, detection of CNVs from WES data has limitations, especially regarding the detection of small CNVs, making a reliable detection difficult. Although we used specific software applications (CNV read-depth analysis tool ExomeDepth; [51]) and manual evaluation of the coverage in highly penetrant genes, we cannot exclude that we have missed deletions or duplications, particularly of single exons or genes. Variants conferring a high lifetime risk are generally considered “diagnostically relevant”, and patients should be informed. However, there is uncertainty and lack of clear guidance with regards to what penetrance, respectively, lifetime risk, a variant should be categorized as relevant for patient counseling. Nevertheless, classifying variants as ‘diagnostically relevant’ is an important category and will become more and more relevant, in view of emerging disease modifying therapies and potentially other actionable choices with regards to family planning and lifestyle choices.

Additionally, we describe patients with established risk variants (TREM2, APOE4 heterozygotes) and rare variants in known risk genes (ABCA7 and SORL1), which have not yet been described in the literature (carriers of well-established and potential new risk variants). Although no reliable assumption about their pathogenicity can be made at this stage, it is plausible that some of them could confer increased risk. The rarity of these missense variants and their incomplete penetrance makes it difficult to assess how they may influence a disease as heterogeneous as dementia. Here, it is also important to evaluate whether they act through a loss or gain of function in order to determine their pathophysiological mechanisms. Testing these variants in larger case–control cohorts is necessary to answer this question. However, it should be mentioned that a reliable statement can only be made if variants are tested in their population-specific cohorts, such as SORL1-p.G1536S, which is almost exclusively present in the Ashkenazi Jewish population.

As expected, carriers of mutations in autosomal dominant genes showed the earliest age of onset and the strongest familial background compared to the other patients. 70% (*n* = 5/7) of them had Goldman scores from 1 to 3, but only 31% (*n* = 5/16) of “risk variant carriers” and 16% (*n* = 5/32) of “no variant carriers” scored in this range. The weaker familial background in these groups may indicate that oligogenic inheritance mechanisms may play a more important role in these patients, as previously suggested for AD [52–55]. In this respect, it is interesting to highlight patient EOD-2, whose disease onset was at 44 years of age, the second youngest in the entire cohort. He carried three disease variants; the likely causal variant MAPT-p.(P636L), the established risk variant TREM2-p.(R62C) and an APOE4/3 genotype. It is tempting to speculate that additive effects may contribute to this exceptionally early disease onset.

Finally, in a more exploratory approach, we aimed to search for potential new risk genes. For this purpose, we compiled a list of 564 genes that were previously considered genetically or functionally related to neurodegeneration (Additional file 1: Table S2). We then prioritized those genes in which variants were found in at least two patients or strongly associated with pathways involved in dementia pathogenesis. We nominate five genes (DCTN1, JIP3/MAPK8IP3, LRRK2, BACE1 and VPS13C) as promising candidates. However, it must be clearly stated that no statistical evidence can be provided for any of these five genes. In addition, due to our selection criteria and the small size of the cohort, very rare variants that possibly contribute to disease risk may have been missed in our analysis. A major problem in identifying new disease genes is the tremendous number of variants and variant combinations in any given individual. At best, statistical proof is possible when the same variant or variant combinations are found repeatedly in different patients. Assuming that new potentially pathogenic variants, as described here, rarely occur, appropriately large cohorts are needed to have a chance of a second finding. We believe that one way of addressing this problem is to provide unabridged sequencing results along with clinical information to the scientific community. Our approach is to deposit all of our exome data in the European Genome-Phenome Archive (EGA), making it accessible to all qualified users. Thereby, we hope to increase the chance that the same gene/variant-hit might be found independently in other well-defined EOD patients, thus confirming new genetic risk variants or variant combinations. We acknowledge that no definite conclusions on pathogenicity of our proposed candidate genes can be drawn from the present study, and we emphasize that the nomination of these candidate genes is intended as a suggestion and impetus for further research.

Altogether, we present 60 EOD patients that have been extensively studied, both genetically and clinically. We show that in only 20%, the underlying disease cause can be explained with high probability by high-penetrant mutations (PSEN1, MAPT, APP, and GRN) and homozygosity of the APOE4/4 genotype These variants are relevant for supporting the diagnosis, patient counseling and, eventually, also for future therapeutic interventions. Carrier status of such genotypes also correlates with early disease onset and, in the case of autosomal dominant variants, the strongest family history in this cohort.

In addition, we present potentially novel risk variants in established and new genes. Our cohort's clinical and genetic data will be made publicly available to allow other researchers to perform an independent analysis. Thus, we hope that potentially new genetic risk variants can be confirmed.

## Materials and methods

### Patients

Participants were recruited from the memory outpatient clinic of the Department of Neurology of the Medical University of Vienna from July 2017 to February 2022. Written informed consent, including publication of data in scientific journals and deposition in scientific databases, was obtained from all participants during the inclusion in two existing registries: Dementia Registry RDA MUV (EK 1323/2018) and the BIOBANK MUV (EK 2195/2016).

The inclusion criterion was the diagnosis of any type of dementia with symptom onset before 65 years. The diagnosis of AD was based on the National Institutes of Health recommendations and the Alzheimer’s Association criteria [56].

Patients were diagnosed with posterior cortical atrophy (PCA) according to the 2017 Consensus classification of posterior cortical atrophy [57].

The diagnosis of behavioral frontotemporal dementia (bvFTD) was based on recommendations of the International Behavioral Variant FTD Criteria Consortium (FTDC) [58]. Diagnosis of primary progressive aphasia (PPA), particularly semantic variant PPA (svPPA), non-fluent/agrammatic variant PPA (nfPPA) and logopenic variant PPA (lpPPA) followed the guidelines of an international group of PPA investigators [59]. Vascular dementia (VD) was diagnosed according to the 1993 recommendation of the National Institute of Neurological Disorders and Stroke and Association Internationale pour la Recherche et l’Enseignement en Neurosciences (NINDS-AIREN) [60]. Diagnosis of mixed dementia followed the 2010 IWG-2 criteria [61]. Lewy Body dementia (LBD) was diagnosed according to the 2017 consensus report of the Dementia with Lewy Bodies Consortium [62]. Amyotrophic lateral sclerosis (ALS) diagnosis was based on the 2020 Goldcoast Criteria [63]. The diagnosis of a corticobasal syndrome (CBS) followed the 2013 criteria by Armstrong et al. [64]. All 60 patients with an objective cognitive decline underwent a thorough standardized diagnostic examination, including physical and neurological evaluation, neuropsychological testing, magnetic resonance imaging (MRI) of the brain and basic laboratory testing. We extended our diagnosis with a biomarker-based approach for a subset of patients. Levels of Aβ_(1–42)_, pTau_(181P)_ and tTau were measured with commercially available enzyme-linked immunosorbent assays (ELISA) (Innotest hTAU-Ag, Innotest phosphoTAU 181p, Innotest beta-Amyloid 1–42) [65, 66].

CSF analyses of established AD biomarkers (Aβ1-42, tTau, pTau) were available in 49 patients, amyloid-PET imaging was performed in 50 patients, and 43 patients underwent both diagnostic methods. Unfortunately, in 4 patients (EOD-11, EOD-25, EOD-33 and EOD-54), it was not possible to perform biomarker testing.

The CSF-biomarker thresholds were as follows: tTau:

-  < 300 pg/ml (21-50y)
-  < 450 pg/ml (51-70y)
-  < 500 pg/ml (71y and older)

pTau _(181P)_: < 61 pg/ml

Aβ_(1–42)_: > 500 pg/ml.

Amyloid positivity was defined by CSF and/or Amyloid-PET Imaging. In cases where both examinations were available or discordant results were obtained, PET determined amyloid status. Family history was categorized by the modified Goldman score [5, 48], indicating a strong to weak familial background of dementia on a scale from 1 to 4, respectively. Patients with unknown family histories were rated as 4.5. All clinical details are shown in Additional file 1: Table S1.

All patients were of European origin.

The project was approved by the Ethics Committee of the Medical University of Vienna (EK 2137/2019) on December 10th, 2019.

### Whole-exome Sequencing (WES)

WES was performed on genomic DNA from 60 EOD index patients using standard protocols followed under stringent quality control, as previously described. Briefly, exomic sequences were enriched in solution and indexed with SureSelect Human All Exon v5 and v6 kits (Agilent Technologies). Sequencing was conducted as 100-bp paired-end runs on an Illumina HiSeq4000 or NovaSeq6000 systems. Raw data processing involved (i) read alignment against the human assembly GRCh37/hg19 using a Burrows–Wheeler-Aligner, (ii) variant calling using Samtools and (iii) variant annotation using Custom-Perl-scripts. At least 95% of targeted exome bases were covered to a depth of 20× or greater in all samples. Copy number variants (CNVs) were called using the Exome Depth algorithm (version 1.1.10) [51]. Called variants included missense, nonsense, splicing, and frameshift variants, together with CNVs. To prioritize variants, minor allele frequency (MAF) in Genome Aggregation Database MAF < 0.01% was applied. First, we explored variants from genes where mutations have been reported to cause autosomal dominant neurodegenerative dementias (PSEN1, PSEN2, APP, GRN, MAPT, VCP, CHMP2B, FUS, TARDBP, TBK1 and PRNP). Additionally, as the, to date, best-known genetic risk factor known for AD, the APOE4 genotype was collected, and APOE4/4 homozygotes were determined as genetically resolved. Second, we selected variants in genes that have already been associated with an increased risk of suffering AD or FTD (ABCA7, SORL1, TREM2). Variants from WES were classified as (i) pathogenic (P), (ii) likely pathogenic (LP), and variants of unknown significance (VUS) according to the ACMG criteria. VUS unrelated to dementia and (likely) benign variants were not reported. Third, we attempted to prioritize potential candidate genes beyond the variants reported in a diagnostic setting. We first generated a candidate gene catalog of 564 genes, including low susceptibility genes from dementia-GWAS studies, genes from WES studies and selected genes strongly associated with neurodegeneration in functional and animal studies (Additional file 1: Table S2). We then searched for rare (MAF < 0.01%) non-synonymous (missense, indel, splice-site, frameshift, nonsense) variants in each exome dataset and intersected those genes with the candidate gene list. Decisive criteria for selecting candidate genes were, among others, the repeated occurrence of the same variant in different patients or the occurrence of different variants in the same gene in different patients. To provide all rare variants to the scientific community, we deposited VCF files of exome datasets of each patient (MAF < 0.01%) to the European Genome-phenome Archive database (EGA) (https://www.ebi.ac.uk/ega/home). Qualified users can receive a password on request to access the data. The complete WES data sets are also available on request by contacting the corresponding author, Elisabeth Stögmann (elisabeth.stoegmann@meduniwien.ac.at).

### C9orf72 testing

Flanking and repeat-primed (RP) PCRs for the sizing of alleles and the detection of C9orf72 (G4C2) hexanucleotide expansion polymorphism were performed using FAM-labelled primers as previously described [67]. Final detection and sizing of PCR products were performed on the ABI 3500 Instrument (Applied Biosystems) using GeneScanTM LIZ600 size standard v.2.0. (Applied Biosystems). To ensure technical and methodological accuracy, a positive control DNA with a proven C9orf72 expansion (> 1500 repeats) was included in the analysis.

### ***RNA sequencing (***Additional file 1***: Fig. S2)***

RNA was isolated from whole blood using the PAXgene® Blood RNA System (BD, Franklin Lakes, USA). According to the manufacturer's recommendations, RNA was extracted using the PAXgene Blood RNA Kit (Quiagen, Venlo, Netherlands). RNA sequencing was performed using the TruSeq Stranded mRNA kit (Illumina, San Diego, USA) for library preparation and a NovaSeq6000 sequencer.

### ***Rab10 phosphorylation analyses of the LRRK2-L2466H variant (***Additional file 1*: ****Fig. S1)***

Monocytes and Neutrophils were isolated from peripheral human blood by immunomagnetic negative selection using the EasySep Human Monocyte/Neutrophil Isolation Kit and Easy 50 EasySep Magnets (STEMCELL Technologies) following the manufacturer’s protocol. Cells were treated with or without the specific LRRK2 kinase inhibitor MLi-2 at 200 nM for 30 min. Quantitative multiplexed immunoblotting was performed as previously described [68] using antibodies against total LRRK2, pSer935 LRRK2, total Rab10, MJFF-pRAB10 (pT73) and GAPDH. Immunoblots were quantified for phospho-Thr73 Rab10/total Rab10 ratio using Odyssey CLx Western Blot imaging.

HEK293 cells were transiently transfected with constructs expressing either wild-type (wt), Flag-LRRK2[G2019S], Flag-LRRK2-R1441G and Flag-LRRK2-R1441G mutant. 24 h post-transfection cells were lysed and analyzed by immunoblotting with the antibodies indicated above [69]. Membranes were developed using Odyssey CLx Western Blot imaging.

### Statistics

Differences in AAO between groups were evaluated using Mann–Whitney U test followed by correction for multiple testing by the Holm-Sidak method. All calculations were performed using GraphPad Prism version 8.0.1

### Supplementary Information

**Additional file 1**. Additional clinical, laboratory and genetic information.

## Data Availability

All data generated or analyzed during this study are included in this published article and its Additional file 1 (containing Table S1: Clinical characteristics of the cohort; Table S2: Curated candidate gene list; Fig. S1: Evaluation of LRRK2-L2466H and Fig. S2: RNA-Seq Analysis of MAPK8IP3). Sequencing data will be available at The European Genome-phenome Archive (EGA) as VCF files. The Data Processing Agreement (DPA) was approved by our legal department and the EGA and data are currently uploaded to the database. Project and sample IDs will be published in this section after completion of data submission.

## References

[1] Hoogmartens J, Cacace R, Van Broeckhoven C (2021). Insight into the genetic etiology of Alzheimer's disease: a comprehensive review of the role of rare variants. Alzheimers Dement (Amst).

[2] Jarmolowicz AI, Chen HY, Panegyres PK (2015). The patterns of inheritance in early-onset dementia: Alzheimer's disease and frontotemporal dementia. Am J Alzheimers Dis Other Demen.

[3] van Duijn CM (1994). Apolipoprotein E4 allele in a population-based study of early-onset Alzheimer's disease. Nat Genet.

[4] Campion D (1999). Early-onset autosomal dominant Alzheimer disease: prevalence, genetic heterogeneity, and mutation spectrum. Am J Hum Genet.

[5] Goldman JS (2005). Comparison of family histories in FTLD subtypes and related tauopathies. Neurology.

[6] Blauwendraat C (2018). The wide genetic landscape of clinical frontotemporal dementia: systematic combined sequencing of 121 consecutive subjects. Genet Med.

[7] Zalar B (2018). Clinical exome sequencing in dementias: a preliminary study. Psychiatr Danub.

[8] Xu Y (2018). The whole exome sequencing clarifies the genotype- phenotype correlations in patients with early-onset dementia. Aging Dis.

[9] Retterer K (2016). Clinical application of whole-exome sequencing across clinical indications. Genet Med.

[10] Richards S (2015). Standards and guidelines for the interpretation of sequence variants: a joint consensus recommendation of the American college of medical genetics and genomics and the association for molecular pathology. Genet Med.

[11] Riaz M (2021). Effect of APOE and a polygenic risk score on incident dementia and cognitive decline in a healthy older population. Aging Cell.

[12] Carmona S (2018). The role of TREM2 in Alzheimer's disease and other neurodegenerative disorders. Lancet Neurol.

[13] Wolfe CM et al. The role of APOE and TREM2 in Alzheimer's Disease-Current understanding and perspectives*.* Int J Mol Sci. 2018; 20(1). 10.3390/ijms20010081 PMC6337314 30587772

[14] De Roeck A, Van Broeckhoven C, Sleegers K (2019). The role of ABCA7 in Alzheimer's disease: evidence from genomics, transcriptomics and methylomics. Acta Neuropathol.

[15] Campion D, Charbonnier C, Nicolas G (2019). SORL1 genetic variants and Alzheimer disease risk: a literature review and meta-analysis of sequencing data. Acta Neuropathol.

[16] Rovelet-Lecrux A (2021). Impaired SorLA maturation and trafficking as a new mechanism for SORL1 missense variants in Alzheimer disease. Acta Neuropathol Commun.

[17] Farrer MJ (2009). DCTN1 mutations in Perry syndrome. Nat Genet.

[18] Konno T (2017). DCTN1-related neurodegeneration: Perry syndrome and beyond. Parkinsonism Relat Disord.

[19] Schroer TA (2004). Dynactin. Annu Rev Cell Dev Biol.

[20] Haenig C (2020). Interactome mapping provides a network of neurodegenerative disease proteins and uncovers widespread protein aggregation in affected brains. Cell Rep.

[21] Miller KG (2017). Keeping neuronal cargoes on the right track: new insights into regulators of axonal transport. Neuroscientist.

[22] Huang SH (2011). JIP3 mediates TrkB axonal anterograde transport and enhances BDNF signaling by directly bridging TrkB with kinesin-1. J Neurosci.

[23] Watt D, Dixit R, Cavalli V (2015). JIP3 activates kinesin-1 motility to promote axon elongation. J Biol Chem.

[24] Platzer K (2019). De novo variants in MAPK8IP3 cause intellectual disability with variable brain anomalies. Am J Hum Genet.

[25] Iwasawa S (2019). Recurrent de novo MAPK8IP3 variants cause neurological phenotypes. Ann Neurol.

[26] Gowrishankar S, Wu Y, Ferguson SM (2017). Impaired JIP3-dependent axonal lysosome transport promotes amyloid plaque pathology. J Cell Biol.

[27] Raghavan NS (2018). Whole-exome sequencing in 20,197 persons for rare variants in Alzheimer's disease. Ann Clin Transl Neurol.

[28] Hsu JL (2021). Genetic study of young-onset dementia using targeted gene panel sequencing in Taiwan. Am J Med Genet B Neuropsychiatr Genet.

[29] Ciani M et al. The missing heritability of sporadic frontotemporal dementia: new insights from rare variants in neurodegenerative Candidate Genes*.* Int J Mol Sci. 2019; 20(16). 10.3390/ijms20163903 PMC6721049 31405128

[30] Fan TS (2016). Clinical heterogeneity of LRRK2 p.I2012T mutation. Parkinsonism Relat Disord.

[31] Wider C, Dickson DW, Wszolek ZK (2010). Leucine-rich repeat kinase 2 gene-associated disease: redefining genotype-phenotype correlation. Neurodegener Dis.

[32] Henderson MX (2019). Alzheimer's disease tau is a prominent pathology in LRRK2 Parkinson's disease. Acta Neuropathol Commun.

[33] Sanchez-Contreras M (2017). Study of LRRK2 variation in tauopathy: progressive supranuclear palsy and corticobasal degeneration. Mov Disord.

[34] Paisan-Ruiz C (2008). Comprehensive analysis of LRRK2 in publicly available Parkinson's disease cases and neurologically normal controls. Hum Mutat.

[35] Steger M et al. Phosphoproteomics reveals that Parkinson's disease kinase LRRK2 regulates a subset of Rab GTPases*.* Elife. 2016; 5. 10.7554/eLife.12813 PMC4769169 26824392

[36] Alessi DR, Sammler E (2018). LRRK2 kinase in Parkinson's disease. Science.

[37] Kalogeropulou AF (2022). Impact of 100 LRRK2 variants linked to Parkinson's disease on kinase activity and microtubule binding. Biochem J.

[38] Fan Y et al. R1441G but not G2019S mutation enhances LRRK2 mediated Rab10 phosphorylation in human peripheral blood neutrophils*.* Acta Neuropathol. 2021. 10.1007/s00401-021-02325-z PMC8357670 34125248

[39] Fan Y (2018). Interrogating Parkinson's disease LRRK2 kinase pathway activity by assessing Rab10 phosphorylation in human neutrophils. Biochem J.

[40] Yan R (2001). The transmembrane domain of the Alzheimer's beta-secretase (BACE1) determines its late Golgi localization and access to beta -amyloid precursor protein (APP) substrate. J Biol Chem.

[41] Mullan M (1992). A pathogenic mutation for probable Alzheimers-disease in the app gene at the N-terminus of beta-amyloid. Nat Genet.

[42] Guerreiro RJ (2010). Genetic screening of Alzheimer's disease genes in Iberian and African samples yields novel mutations in presenilins and APP. Neurobiol Aging.

[43] Zhou L (2011). Amyloid precursor protein mutation E682K at the alternative beta-secretase cleavage beta-site increases A beta generation. EMBO Mol Med.

[44] Li P (2019). Epigenetic dysregulation of enhancers in neurons is associated with Alzheimer's disease pathology and cognitive symptoms. Nat Commun.

[45] Lesage S (2016). Loss of VPS13C function in autosomal-recessive parkinsonism causes mitochondrial dysfunction and increases PINK1/Parkin-dependent mitophagy. Am J Hum Genet.

[46] Smolders S (2021). Contribution of rare homozygous and compound heterozygous VPS13C missense mutations to dementia with Lewy bodies and Parkinson's disease. Acta Neuropathol Commun.

[47] Zhao Z (2020). UK biobank whole-exome sequence binary phenome analysis with robust region-based rare-variant test. Am J Hum Genet.

[48] Beck J (2008). A distinct clinical, neuropsychological and radiological phenotype is associated with progranulin gene mutations in a large UK series. Brain.

[49] Langlois CM (2019). Alzheimer's prevention initiative generation program: development of an APOE genetic counseling and disclosure process in the context of clinical trials. Alzheimers Dement (N Y).

[50] Cummings J et al. Lecanemab: appropriate use recommendation*s.* J Prev Alzheimer's Dis. 2023. 10.14283/jpad.2023.30 PMC10313141 37357276

[51] Plagnol V (2012). A robust model for read count data in exome sequencing experiments and implications for copy number variant calling. Bioinformatics.

[52] Escott-Price V (2015). Common polygenic variation enhances risk prediction for Alzheimer's disease. Brain.

[53] Altmann A (2020). A comprehensive analysis of methods for assessing polygenic burden on Alzheimer's disease pathology and risk beyond APOE. Brain Commun.

[54] Zhang Q (2020). Risk prediction of late-onset Alzheimer's disease implies an oligogenic architecture. Nat Commun.

[55] Fulton-Howard B (2021). Greater effect of polygenic risk score for Alzheimer's disease among younger cases who are apolipoprotein E-epsilon4 carriers. Neurobiol Aging.

[56] Jack CR (2018). NIA-AA research framework: toward a biological definition of Alzheimer's disease. Alzheimers Dement.

[57] Crutch SJ (2017). Consensus classification of posterior cortical atrophy. Alzheimers Dement.

[58] Rascovsky K (2011). Sensitivity of revised diagnostic criteria for the behavioural variant of frontotemporal dementia. Brain J Neurol.

[59] Gorno-Tempini ML (2011). Classification of primary progressive aphasia and its variants. Neurology.

[60] Román GC (1993). Vascular dementia: diagnostic criteria for research studies. Report of the NINDS-AIREN international workshop. Neurology.

[61] Dubois B (2014). Advancing research diagnostic criteria for Alzheimer's disease: the IWG-2 criteria. Lancet Neurol.

[62] McKeith IG (2017). Diagnosis and management of dementia with Lewy bodies: fourth consensus report of the DLB consortium. Neurology.

[63] Shefner JM (2020). A proposal for new diagnostic criteria for ALS. Clin Neurophysiol.

[64] Armstrong MJ (2013). Criteria for the diagnosis of corticobasal degeneration. Neurology.

[65] Vanderstichele H (2000). Standardization of measurement of beta-amyloid(1–42) in cerebrospinal fluid and plasma. Amyloid.

[66] Vanmechelen E (2000). Quantification of tau phosphorylated at threonine 181 in human cerebrospinal fluid: a sandwich ELISA with a synthetic phosphopeptide for standardization. Neurosci Lett.

[67] Renton AE (2011). A hexanucleotide repeat expansion in C9ORF72 is the cause of chromosome 9p21-linked ALS-FTD. Neuron.

[68] Fan Y (2021). R1441G but not G2019S mutation enhances LRRK2 mediated Rab10 phosphorylation in human peripheral blood neutrophils. Acta Neuropathol.

[69] Wang X (2021). Understanding LRRK2 kinase activity in preclinical models and human subjects through quantitative analysis of LRRK2 and pT73 Rab10. Sci Rep.

