# Correction: Comprehensive genetic screening of early-onset dementia patients in an Austrian cohort-suggesting new disease-contributing genes

**DOI:** 10.1186/s40246-023-00525-0

**Published:** 2023-08-28

**Authors:** Sara Silvaieh, Theresa König, Raphael Wurm, Tandis Parvizi, Evelyn Berger-Sieczkowski, Stella Goeschl, Christoph Hotzy, Matias Wagner, Riccardo Berutti, Esther Sammler, Elisabeth Stögmann, Alexander Zimprich

**Affiliations:** 1 Department of Neurology, Medical University of Vienna, Vienna, Austria; 2 Comprehensive Center for Clinical Neurosciences and Mental Health, Medical University of Vienna, Vienna, Austria; 3 Institute of Human Genetics, School of Medicine, Technical University of Munich, Munich, Germany; 4 Institute of Neurogenomics, Helmholtz Centrum, Munich, Germany; 5 Molecular and Clinical Medicine, Ninewells Hospital and Medical School, University of Dundee, Dundee, DD1 9SY UK; 6 Medical Research Council Protein Phosphorylation and Ubiquitylation Unit, School of Life Sciences, University of Dundee, Dundee, DD1 5EH UK

**Correction to: Human Genomics (2023) 17:55** **https://doi.org/10.1186/s40246-023-00499-z**

Following publication of the original article [1], the authors reported an error in Table 1. The correct Table 1 has been provided in this Correction.

**Table 1:** Basic clinical and genetic characteristics of all 60 EOD patients

ID	Diagnosis	AAO (years)	Sex	FH	APOE	Gene	Variant	Position	Transcript	CADD	ClinVar	Significance for disease
EOD-1	**AD**	**54**	**f**	**3**	**E2/E3**	***PSEN1***	**c.356C > T; p.T119I**	**chr14:73640291-73640291**	**NM_000021.3**	**24.4**	**LP**	**Relevant for diagnosis**
EOD-2	**bvFTD**	**44**	**f**	**1**	**E4/E3**	***MAPT***	**c.1907C > T; p.P636L**	**chr17:44087755-44087755**	**NM_001123066.3**	**34.0**	**P**	**Relevant for diagnosis**
						*TREM2*	c.184G > A; p.R62C	chr6:41129208-41129208	NM_001271821.1	25.5	n.r	Risk modifier
						*APOE*						Risk modifier
EOD-3	AD	45	f	2	E3/E3							
EOD-4	AD	51	f	4	E4/E3	*APOE*						Risk modifier
EOD-5	nfPPA	58	f	2	E3/E2							
EOD-6	AD	56	f	3	E3/E3							
EOD-7	AD/PCA	56	f	4	E3/E3							
EOD-8	bvFTD	56	m	4	E3/E3	*BACE1*	c.1427T > C; p.M476T	chr11:117160361-117160361	NM_012104.3	26.4	n.r	Unknown
						*VPS13C*	c.9757A > G; p.S3253G	chr15:62173781-62173781	NM_020821.2	29.5	n.r	Unknown
EOD-9	AD	55	f	3,5	E4/E3	*APOE*						Risk modifier
EOD-10	AD	58	f	3,5	E3/E3							
EOD-11	AD	63	m	4	E3/E3							
EOD-12	mixed dementia (AD + VD)	55	m	3,5	E4/E3	*APOE*						Risk modifier
EOD-13	AD	61	m	4,5	E3/E3							
EOD-14	AD/lpPPA	61	m	4	E4/E3	*APOE*						Risk modifier
						*VPS13C*	c.4300C > T; p.V1434I	chr15:62244179-62244179	NM_020821.2	24.8	n.r	Unknown
EOD-15	nfPPA	64	m	2	E3/E3	*DCTN1*	c.2218C > T; p.E740K	chr2:74594514-74594514	NM_004082.4	24.0	n.r	Unknown
EOD-16	AD	56	f	4	E3/E3							
EOD-17	AD (PD)	60	m	1	E4/E3	*APOE*						Risk modifier
						*MAPK8IP3*	g.chr16:1816528 A > G; c.2817-2A > G	chr16:1816528-1816528	NM_015133.3	22.3	n.r	Unknown
EOD-18^a^	**AD**	**47**	**m**	**4**	**E3/E3**	***APP***	**g.chr.21:(?_26958019)-(27852747_?)dup**				**P**	**Relevant for diagnosis**
						*ABCA7*	c.2914C > T; p.P972S	chr19:1051537-1051537	NM_019112.3	25.3	n.r	Potential risk modifier
EOD-19	**AD**	**51**	**m**	**1**	**E3/E3**	***APP***	**g.chr.21:(?_27253981)-(27542937_?)dup**				**P**	**Relevant for diagnosis**
EOD-19 (2)^b^	**AD**	**47**	**m**	**1**	**E3/E3**	***APP***	**g.chr.21:(?_27253981)-(27542937_?)dup**				**P**	**Relevant for diagnosis**
EOD-20	AD	57	m	4,5	E3/E3	*LRRK2*	c.7397T > A; p.L2466H	chr12:40760814-40760814	NM_198578.3	25.7	VUS	Unknown
EOD-21	**CAA**	**54**	**m**	**3,5**	**E4/E4**	***APOE***						**Relevant for diagnosis**
EOD-22	**AD**	**49**	**m**	**4**	**E4/E4**	***APOE***						**Relevant for diagnosis**
EOD-23	**AD**	**36**	**f**	**1**	**E3/E3**	***PSEN1***	**c.617G > A; p.G206D**	**chr14:73659420-73659420**	**NM_000021.3**	**31.0**	**P**	**Relevant for diagnosis**
EOD-24	**AD**	**53**	**m**	**3,5**	**E4/E4**	***APOE***						**Relevant for diagnosis**
EOD-25	**AD**	**51**	**f**	**3,5**	**E4/E4**	***APOE***						**Relevant for diagnosis**
EOD-26	AD	56	f	4	E3/E3	*DCTN1*	c.2980G > C; p.P994A	chr2:74590268-74590268	NM_023019.3	17.3	VUS	Unknown
						*MAPK8IP3*	c.2087G > A; p.R696H	chr16:1814180-1814180	NM_015133.3	31.0	n.r	Unknown
EOD-27	AD	57	f	4	E4/E3	*APOE*						Risk modifier
EOD-28	AD	54	m	4	E3/E3							
EOD-29	AD	54	m	4	E3/E3							
EOD-30	AD	64	m	4	E3/E3							
EOD-31	mixed dementia (AD + VD)	58	m	3,5	E3/E3							
EOD-32	FTD/svPPA	61	m	4	E3/E3							
EOD-33	AD	62	f	4,5	E4/E3	*APOE*						Risk modifier
						*DCTN1*	c.521G > A; p.S174L	chr2:74598788-74598788	NM_004082.4	24.4	VUS	Unknown
EOD-34	AD	59	f	2	E4/E3	*APOE*						Risk modifier
EOD-35	AD	55	m	3,5	E4/E3	*APOE*						Risk modifier
EOD-36^c^	AD	64	m	2	E4/E3	*TREM2*	c.140G > A; p.R47H	chr6:41129252-41129252	NM_018965.3	9.7	LB	Risk modifier
						*APOE*						Risk modifier
EOD-37	AD	52	f	3,5	E3/E3	*LRRK2*	c.7397T > A; p.L2466H	chr12:40760814-40760814	NM_198578.3	25.7	VUS	Unknown
EOD-38	AD	52	f	3,5	E4/E3	*APOE*						Risk modifier
EOD-39	AD	63	f	3	E4/E3	*APOE*						Risk modifier
EOD-40	AD	55	f	4	E4/E3	*APOE*						Risk modifier
EOD-41	AD	58	m	3,5	E3/E3							
EOD-42	AD	39	m	4	E3/E2							
EOD-43	AD	63	m	4	E3/E3	*VPS13C*	c.3148A > G; p.I1050V	chr15:62256964-62256964	NM_020821.2	0.001	VUS	Unknown
EOD-44	AD/lpPPA	58	f	3,5	E3/E3	*SORL1*	c.3014T > G; p.M1005R	chr11:121430331-121430331	NM_003105.5	27.9	n.r	Potential risk modifier
EOD-45	AD	65	m	4	E3/E3							
EOD-46	CBS + AD	51	f	3,5	E3/E3	*SORL1*	c.4606G > A; p.G1536S	chr11:121474988-121474988	NM_003105.5	25.2	B	Risk modifier
EOD-47	AD	54	f	4	E3/E3							
EOD-48	bvFTD	57	m	4	E3/E3							
EOD-49	FTD/nfPPA + ALS	58	m	4	E3/E3	*TBK1*	c.986T > C; p.L276P	chr12:64875636-64875636	NM_013254.3		n.r	Potential risk modifier
						*VPS13C*	c.7436T > C; p.I2429T	chr15:62212307-62212307	NM_020821.2		n.r	Unknown
EOD-50	**FTD (bvFTD + nfPPA)**	**55**	**f**	**3,5**	**E4/E3**	***PGRN***	c.328C > T; p.R110*	**chr17:42427098-42427098**	**NM_002087.3**	**29.4**	**P**	**Relevant for diagnosis**
						*APOE*						Risk modifier
EOD-51	FTD/svPPA	62	f	4	E3/E3							
EOD-52	AD	57	m	4	E4/E3	*APOE*						Risk modifier
EOD-53	**AD**	**57**	**m**	**4**	**E4/E4**	***APOE***						**Relevant for diagnosis**
						*LRRK2*	c.7377G > A; p.M2459I	chr12:40758839-40758839	NM_198578.3	17.7	n.r	Unknown
EOD-54	AD	59	m	1	E4/E3	*APOE*						Risk modifier
EOD-55	AD	49	m	4	E3/E3							
EOD-56	AD	61	m	3,5	E3/E3							
EOD-57	AD/lpPPA	57	f	4	E3/E3							
EOD-58	AD + VD	64	f	3	E3/E3	*DCTN1*	c.823C > T; p.R141C	chr2:74598126-74598126	NM_004082.3	29.3	VUS	Unknown
EOD-59	bvFTD	52	m	4	E4/E3	*APOE*						Risk modifier
EOD-60	**AD**	**49**	**f**	**3**	**E3/E3**	***APP***	**c.2092G > A; p.V586I**	**chr21:27264096**	**NM_201413.3**	**28.2**	**P**	**Relevant for diagnosis**

**a**, EOD-18: The APP duplication of was confirmed to be 'de novo'. Both parents did not show this duplication

**b**, EOD-19 (2) is the brother of EOD19. He was also affected by AD and carrier of the same duplication. EOD 19 (2) was not included in the analyses of AAO and FH

**c**, EOD-36: ClinVar assessment of TREM2 p.R47H of LB (likely benign) refers to Nasu-Hakola disease. However, p.R47H is an established risk variant for dementia (Ref. 15)

The original article [1] has been corrected.
