# Supplementary material for: Comprehensive genetic screening of early-onset dementia patients in an Austrian cohort-suggesting new disease-contributing genes
Source: Hum Genomics. 2023 Jun 17;17:55. doi: 10.1186/s40246-023-00499-z (PMC10276391; doi:10.1186/s40246-023-00499-z)
Supplement: Supplementary file 1 — Additional file 1. Additional clinical, laboratory and genetic information. [file 40246_2023_499_MOESM1_ESM.pdf]

Additional clinical, laboratory and genetic information: Comprehensive genetic screening of early-onset dementia patients in an Austrian Cohort-Suggesting new disease-contributing genes

Sara Silvaieh[1,2†], Theresa König[1,2†], Raphael Wurm[1,2], Tandis Parvizi[1,2], Evelyn Berger-Sieczkowski[1,2], Stella Goeschl[1,2], Christoph Hotzy[1,2], Matias Wagner[3,4], Riccardo Berutti[3], Esther Sammler[5,6], Elisabeth Stögmann[1,2*] and Alexander Zimprich[1,2]

[1] Department of Neurology, Medical University of Vienna, Vienna, Austria; sara.silvaieh@meduniwien.ac.at (S.S.); theresa.koenig@meduniwien.ac.at (T.K.); raphael.wurm@meduniwien.ac.at (R.W.); tandis.parvizi@meduniwien.ac.at (T.P.); evelyn.berger-sieczkowski@meduniwien.ac.at (E.B.-S.); stella.goeschl@meduniwien.ac.at (S.G.); christoph.hotzy@meduniwien.ac.at (C.H.); elisabeth.stoegmann@meduniwien.ac.at (E.S.); alexander.zimprich@meduniwien.ac.at (A.Z.)

[2] Comprehensive Center for Clinical Neurosciences & Mental Health, Medical University of Vienna, Vienna, Austria; sara.silvaieh@meduniwien.ac.at (S.S.); theresa.koenig@meduniwien.ac.at (T.K.); raphael.wurm@meduniwien.ac.at (R.W.); tandis.parvizi@meduniwien.ac.at (T.P.); evelyn.berger-sieczkowski@meduniwien.ac.at (E.B.-S.); stella.goeschl@meduniwien.ac.at (S.G.); christoph.hotzy@meduniwien.ac.at (C.H.); elisabeth.stoegmann@meduniwien.ac.at (E.S.); alexander.zimprich@meduniwien.ac.at (A.Z.)

[3] Institute of Human Genetics, School of Medicine, Technical University of Munich, Munich, Germany; matias.wagner@mri.tum.de (M.W.); riccardo.berutti@mri.tum.de (R.B.)

[4] Institute of Neurogenomics, Helmholtz Centrum, Munich, Germany; matias.wagner@mri.tum.de (M.W.)

[5] Molecular and Clinical Medicine, Ninewells Hospital and Medical School, University of Dundee, Dundee, DD1 9SY, UK; e.m.sammler@dundee.ac.uk

[6] Medical Research Council Protein Phosphorylation and Ubiquitylation Unit, School of Life Sciences, University of Dundee, Dundee DD1 5EH, UK

† Authors contributed equally to this work

* Correspondence: elisabeth.stoegmann@meduniwien.ac.at; Tel.: +43140400-31170

Additional file 1: Fig. S1

A. LRRK2 dependent Rab10^{Thr73} phosphorylation (pRab10) in human peripheral blood neutrophils and monocytes of the LRRK2 p.(L2466H) variant.

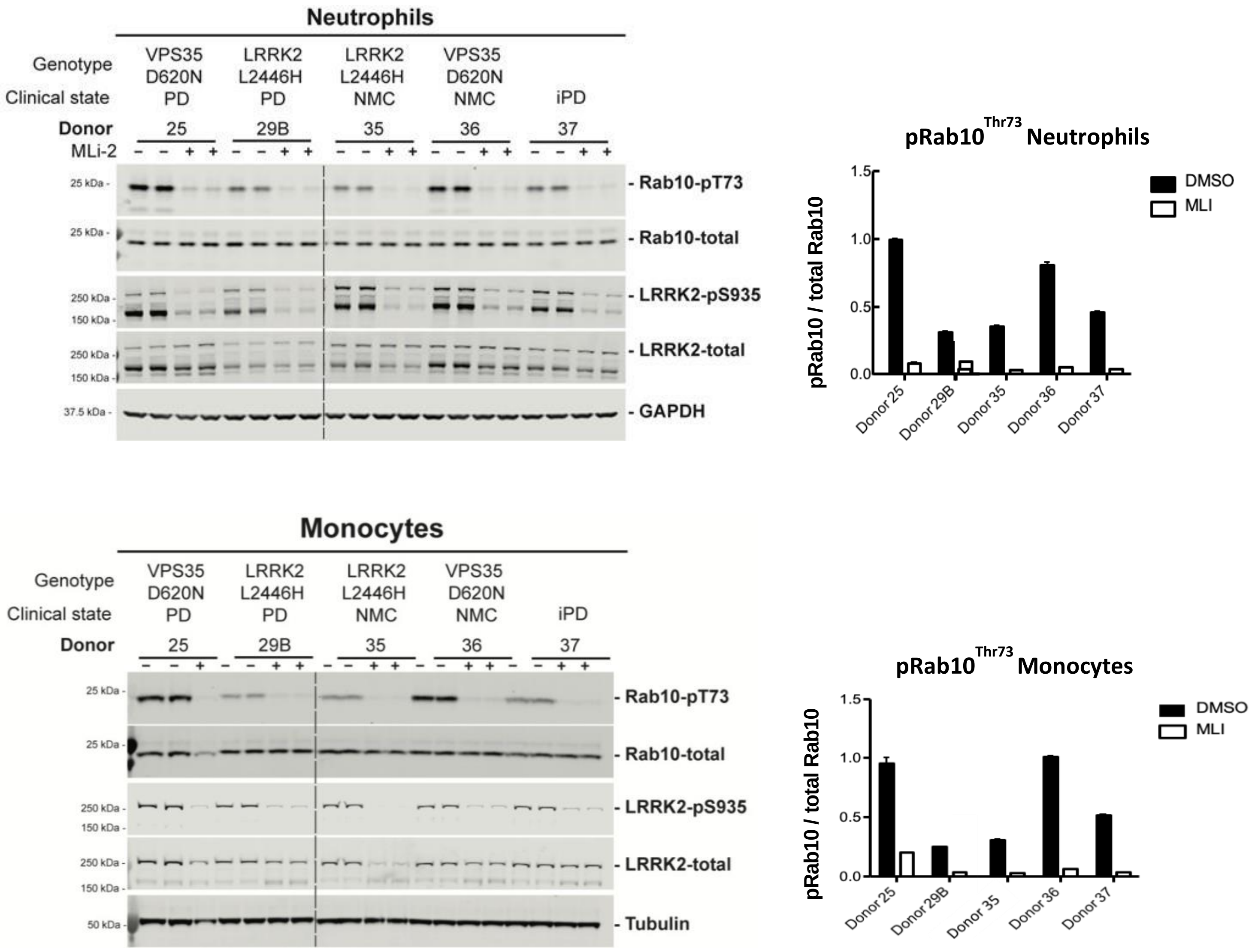

B. LRRK2 dependent Rab10^{Thr73} phosphorylation of the LRRK2 p.(L2466H) variant in a Flag-LRRK2 overexpression system in HEK293 cells.

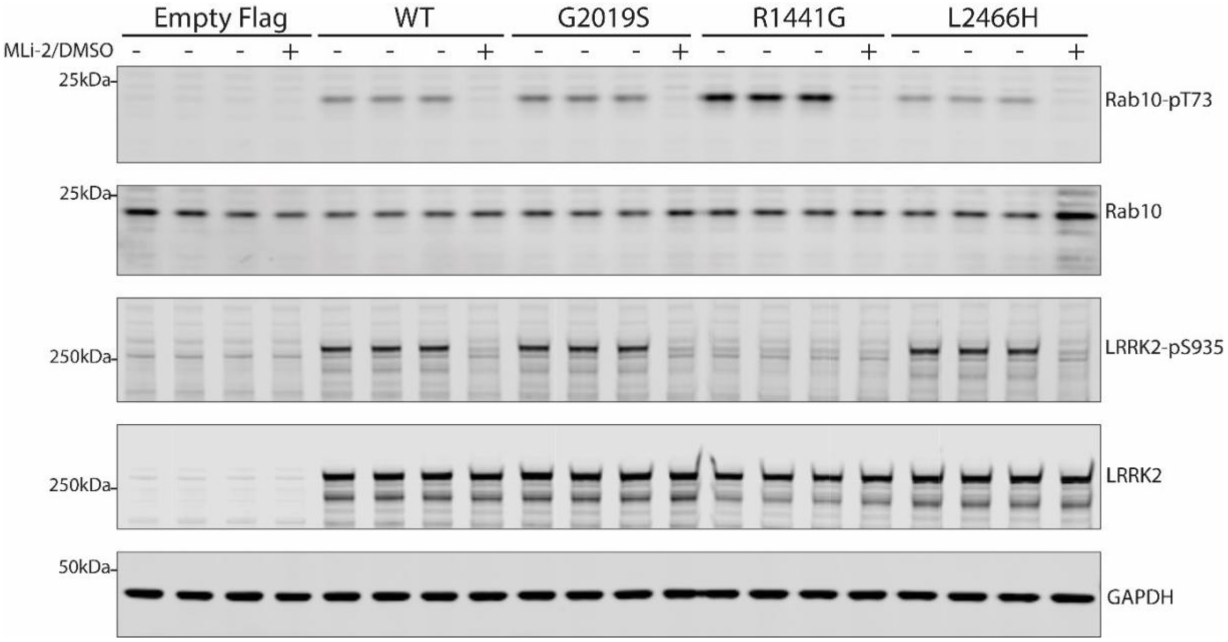

A. **The LRRK2 p.(L2466H) variant does not increase Rab10^{Thr73} phosphorylation in neutrophils and monocytes**. Neutrophiles and Monocytes were isolated from patient EOD-20 (donor 29b), from his healthy mother (donor 35), from one idiopathic PD patient (donor37) serving here as negative control and from two carriers of a VPS35-D620N mutation (one manifesting PD patient, donor 25)and one non-manifesting carrier, donor 36). The VPS35-D620N variant is known to strongly increase rab10 phosphorylation, which serves here as positive control . Monocyte and Neutrophil isolation, characterisation, treatments, and lysis from peripheral human blood were performed by immunomagnetic negative selection using the EasySep Human Monocyte Isolation Kit (STEMCELL Technologies, Cat# 19359) and Easy 50 EasySep Magnets (STEMCELL Technologies, Cat# 18002) following the manufacturer's protocol.

B. **The LRRK2 p.(L2466H) variant does not increase Rab10^{Thr73} phosphorylation in a Flag-LRRK2 overexpression system in HEK293 cells**. HEK293 cells were transiently transfected with constructs expressing either wild-type, Flag-LRRK2[G2019S], Flag-LRRK2-R1441G and Flag-LRRK2-L2466H mutant.LRRK2-R1441G is known to strongly increase rab10 phosphorylation and serves here as a positive control. 24 hr post-transfection cells were lysed and analyzed by immunoblotting with the indicated antibodies.
In both experimentalproceduress A and B, cells were treated with or without 200 nM MLi-2 for 30 min. MLi-2 is a specific LRRK2 kinase inhibitor[1]. Suppression of pRab10 with MLi-2 confirms specificLRRK2-mediatedd increase of kinase function. Immunoblotting was performed as recently described using antibodies against total LRRK2, pSer935 LRRK2, total Rab10, MJFF-pRAB10 (pThr73) and GAPDH [2] [3]. Immunoblots were quantified for phospho-Thr73 Rab10/total Rab10 ratio, using Odyssey CLx Western Blot imaging. (right panel).

Additional file 1: Fig. S2: RNA-Seq Analysis of *MAPK8IP3* of EOD-17

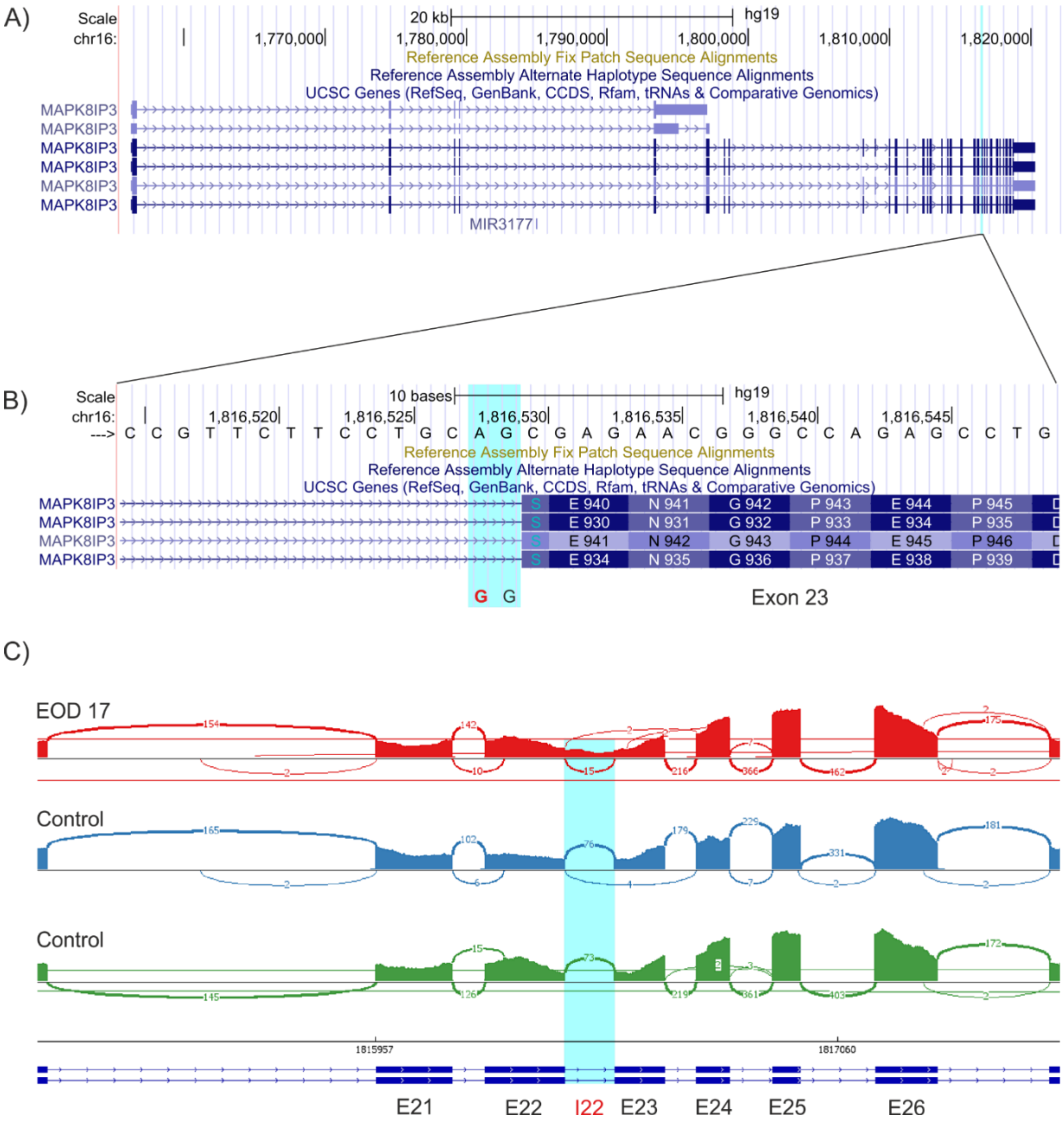

Whole RNA-Seq analysis of peripheral blood monocytes (PBMCs) revealed aberant splicing of MAPK8IP3 in patient EOD-17.

- **A)** Screenshot of the UCSC Genome Browser, showing different transcripts of the *MAPK8IP3* gene. The blue background highlights the area of the variant (g.chr16:1816528 A>G; c. 2817-2A>G, NM_015133) at the Intron 22-Exon-23 junction
- **B)** "Zoomed in" area of the variant location. The splice site acceptor sequence AG is changed to GG (blue background).
- **C)** Sashimi plot revealed that the variant resulted in aberrant splicing between Exon22 and Exon 23 with retention of intron 22 sequences in the patient compared to two healthy controls (blue background).

References:

1. Fell, M.J., et al., *MLi-2, a Potent, Selective, and Centrally Active Compound for Exploring the Therapeutic Potential and Safety of LRRK2 Kinase Inhibition.* J Pharmacol Exp Ther, 2015. **355**(3): p. 397-409.
2. Fan, Y., et al., *Interrogating Parkinson's disease LRRK2 kinase pathway activity by assessing Rab10 phosphorylation in human neutrophils.* Biochem J, 2018. **475**(1): p. 23-44.
3. Fan, Y., et al., *R1441G but not G2019S mutation enhances LRRK2 mediated Rab10 phosphorylation in human peripheral blood neutrophils.* Acta Neuropathol, 2021. **142**(3): p. 475-494.

Additional file 1: Table S1

Detailed clinical characteristics of all 60 EOD patients

No.	Sex	Diagnosis (other relevant diagnoses)	AAO (y)	Symptoms	MRI	Other biomarkers	Family History (Goldman Score)
EOD-1	f	AD	54	1. spatial orientation<br>2. concentration	1.mild biparietal atrophy<br>2.mild white matter hyperintensities<br>3.iron deposition in the basal ganglia, Ncl. Ruber, substantia nigra, dentate nucleus (bilateral)	1. CSF: missing<br>2. FDG-PET: hypometabolism in the right parietotemporal lobe<br>3. Amyloid-PET: cortical amyloid load	Father: unspecified dementia (AAO 60)<br>Goldman Score: 3
EOD-2	f	bvFTD	44	1. episodic memory<br>2. word retrieval<br>3. behavioural symptoms (aggressive behaviour, apathy)	1.significant supratentorial atrophy with symmetric emphasis on the frontotemporal lobes and mild atrophy of the hippocampal and posterior cingulate regions bilateral	1. CSF: missing<br>2. FDG-PET: missing<br>3. Amyloid-PET: no amyloid load	1. Grandmother: unspecified dementia (AAO unknown, died at the age of 70)<br>2. Mother: unspecified dementia (AAO unknown, died at the age of 47)<br>3. Sister: EOAD (AAO 47).<br>Goldman Score: 1
EOD-3	f	AD	45	1. episodic memory<br>2. concentration	1. no atrophy<br>2. mild white matter hyperintensities	1. CSF:<br>Aβ: 434pg/ml<br>pTau: 123pg/ml<br>Tau: 1267pg/ml<br>2. FDG-PET: hypometabolism in the left frontal, temporal, parietal and to a lesser extent in the right parietal cortex, thinning of the right temporolateral cortex<br>3. Amyloid-PET: cortical amyloid load	1.Father: uncertain dementia (AAO unknown)<br>2.Grandfather: unspecified dementia (AAO unknown)<br>3. mother: unspecified dementia (AAO unknown)<br>4. sister: FTD (53y)<br>Goldman Score: 2
EOD-4	f	AD	51	1. episodic memory<br>2. dyscalculia<br>3. spatial orientation	1.Mild supratentorial atrophy, no dementia-specific atrophy pattern	1. CSF:<br>Aβ: 171pg/ml<br>pTau: 68pg/ml<br>Tau: 260pg/ml<br>2. FDG-PET: hypometabolism in both parietotemporal lobes and in both precuneus regions (to a lesser extent in both frontal lobes)<br>3. Amyloid-PET: missing	negative<br>Goldman Score: 4

No.	Sex	Diagnosis (other relevant diagnoses)	AAO (y)	Symptoms	MRI	Other biomarkers	Family History (Goldman Score)
EOD-5	f	FTD/nfPPA	58	1. word retrieval<br>2. episodic memory<br>3. behavioural symptoms (disinhibition)	1. Global atrophy<br>2. mild white matter hyperintensities	1. CSF:<br>Aβ: 457pg/ml<br>pTau: 54pg/ml<br>Tau: 240pg/ml<br>2. FDG-PET: hypometabolism in both temporal lobes<br>3. Amyloid-PET: normal findings	1. Father: unspecified dementia (AAO <65y)<br>2. sister: unspecified dementia (AAO<65)<br>Goldman Score: 2
EOD-6	f	AD	56	1. episodic memory<br>2. concentration<br>3. word retrieval	1. no atrophy<br>2. mild white matter hyperintensities	1. CSF:<br>Aβ: 342pg/ml<br>pTau: 167pg/ml<br>Tau: 1501pg/ml<br>2. FDG-PET: hypometabolism in the right frontal and both parietal lobes<br>3. Amyloid-PET: cortical amyloid load with emphasis on the frontal and parieto-occipital lobes, both precuneus regions and caudate nucleus	1.grandmother: unspecified dementia (AAO 55)<br>Goldman Score: 3
EOD-7	f	AD/PCA	56	1. visuconstructive deficits<br>2. articulation<br>3. word retrieval	1. Temporoparietal atrophy<br>2. mild white matter hyperintensities	1. CSF:<br>Aβ: 251pg/ml<br>pTau: 85pg/ml<br>Tau: 978pg/ml<br>2. FDG-PET: hypometabolism in the left parietotemporal lobe, in both precuneus regions and in both occipitolateral lobes<br>3. Amyloid-PET: cortical amyloid load in both frontal lobes, partially in both parietotemporal lobes, in both precuneus regions and caudate nucleus	negative<br>Goldman Score: 4
EOD-8	m	bvFTD	56	1. behavioural symptoms (social withdrawal)<br>2. increased appetite<br>3. episodic memory<br>4. word retrieval<br>5. spatial orientation	1. bilateral hippocampal atrophy, mild supratentorial atrophy<br>2. small chronic infarctions and mild white matter hyperintensities	1. CSF:<br>Aβ: 1051pg/ml<br>pTau: 65pg/ml<br>Tau: 345pg/ml<br>2. FDG-PET: mild hypometabolism in the frontoparietal region<br>3. Amyloid-PET normal finding	Negative<br>Goldman Score: 4

No.	Sex	Diagnosis (other relevant diagnoses)	AAO (y)	Symptoms	MRI	Other biomarkers	Family History (Goldman Score)
EOD-9	f	AD	55	1. spatial orientation	1.mild diffuse atrophy	1. CSF:<br>Aβ: 512pg/ml<br>pTau: 61pg/ml<br>Tau: 349pg/ml<br>2. FDG-PET: hypometabolism in the right precuneus region, right temporal, parietal and occipital lobe<br>3. Amyloid-PET: missing	1.Mother: unspecified dementia (AAO 83)<br>Goldman Score: 3.5
EOD-10	f	AD	58	1. episodic memory<br>2. spatial orientation,<br>3. word retrieval	1. significant supratentorial subcortical atrophy,<br>2. mild white matter hyperintensities	1. CSF:<br>Aβ: 356pg/ml<br>pTau: 80pg/ml<br>Tau: 1105pg/ml<br>2. FDG-PET: hypometabolism in both parietal lobes reaching the temporal cortex, hypometabolism in the left occipital and frontal lobes and left precuneus region<br>3. Amyloid-PET: global cortical amyloid load (emphasis on both precuneus regions and both frontal and temporal lobes)	1.Mother: unspecified dementia (AAO>65)<br>Goldman Score: 3.5
EOD-11	m	AD	63	1. word retrieval<br>2. spatial orientation<br>3. behavioural symptoms (aggressive behaviour)	MRI not possible due to cardiac pacemaker<br>CCT:<br>1. Supratentorial atrophy, bilateral hippocampal atrophy	1. CSF: missing<br>2. FDG-PET: hypometabolism in both parietotemporal and frontal lobes<br>3. Amyloid-PET: missing	negative<br>Goldman Score: 4
EOD-12	m	Mixed dementia (AD+VD)	55	1. episodic memory<br>2. spatial orientation	1. cortical atrophy in the right parietal and central region<br>2. moderate white matter hyperintensities<br>3. infarction in the right frontal and parietal lobe and left parietal lobe<br>3. pineal gland cyst	1. CSF:<br>Aβ: 480pg/ml<br>pTau: 104pg/ml<br>Tau: 1396 pg/ml<br>2. FDG-PET: missing<br>3. Amyloid-PET: cortical amyloid load in frontobasal and precuneus regions and basal ganglia	1.Father: unspecified dementia (AAO 81)<br>Goldman Score: 3.5

No.	Sex	Diagnosis (other relevant diagnoses)	AAO (y)	Symptoms	MRI	Other biomarkers	Family History (Goldman Score)
EOD-13	m	AD	61	1. episodic memory<br>2. concentration<br>3. spatial orientation	1. atrophy of both mesiotemporal lobes and hippocampus regions (mainly on the right)<br>2. mild white matter hyperintensities	1. CSF: missing<br>2. FDG-PET: hypometabolism in both temporoparietal and frontal lobes (emphasis on the right side), hypometabolism in the right occipital lobe<br>3. Amyloid-PET: cortical amyloid load in both frontal lobes, anterior and posterior cingulate, both precuneus regions, insula and both temporal lobes	unknown<br>Goldman Score: 4.5
EOD-14	m	AD/lpPPA	61	1. word retrieval	1. diffuse global atrophy, accentuated on the medial temporal lobe<br>2. mild white matter hyperintensities	1. CSF:<br>Aβ: 665pg/ml<br>pTau: 17pg/ml<br>Tau: 1390 pg/ml<br>2. FDG-PET: hypometabolism in both cortical frontal, temporal and parietal lobes (emphasis on the left side) and in the left occipital lobe<br>3. Amyloid-PET: cortical amyloid load	negative<br>Goldman Score: 4
EOD-15	m	FTD/nfPPA	64	1. word retrieval<br>2. episodic memory<br>3. spatial orientation	1.diffuse global atrophy, accentuated on the medial temporal lobes	1. CSF: missing<br>2. FDG-PET: cortical hypometabolism in both mesiotemporal areas<br>3. Amyloid-PET: normal findings	1. Sister: AD (AAO 70)<br>2. Cousin: AD (AAO not known, died at the age of 70)<br>Goldman Score: 2
EOD-16	f	AD	56	1. ideomotor apraxia<br>2. episodic memory<br>3. concentration<br>4. word-retrieval	1. mild cerebral atrophy accentuated on the right hemisphere, atrophy of the basal ganglia (particularly caudate nucleus)<br>2. moderate white matter hyperintensities	1. CSF:<br>Aβ: 237pg/ml<br>pTau: 77pg/ml<br>Tau: 598 pg/ml<br>2. FDG-PET: hypometabolism in both frontal, parietal and temporal lobes (accentuated on the right side)<br>3. Amyloid-PET: cortical amyloid load	negative<br>Goldman Score: 4

No.	Sex	Diagnosis (other relevant diagnoses)	AAO (y)	Symptoms	MRI	Other biomarkers	Family History (Goldman Score)
EOD-17	m	AD (PD)	60	1. episodic memory<br>2. spatial orientation<br>3. ideomotor apraxia<br>4. tremor, bradykinesia, rigidity, postural instability	1. diffuse global atrophy accentuated on the left side and supratentorial, no specific dementia pattern	1. CSF: missing<br>2. FDG-PET: cortical hypometabolism in both parietal, temporal (accentuated on the left side), fronto-and occipitolateral lobes as well as in both precuneus regions.<br>3. Amyloid-PET: missing<br>4. DaT-SPECT: reduced uptake in the left putamen and caudate nucleus.<br>- ApoE4/E3	1. Mother: unspecified dementia (AAO 70)<br>2. 3(out of 5) maternal aunts: unspecified dementia (AAO 75 and >80)<br>3. grandmother: unspecified dementia (AAO unknown)<br>4. cousin: AD (AAO 60)<br>Goldman Score: 1
**EOD-18**	**m**	**AD (Pituitary adenoma)**	**47**	**1. episodic memory<br>2. seizures**	**1. no atrophy<br>2. mild white matter hyperintensities<br>3. Pituitary adenoma**	**1. CSF:<br>Aβ: 218pg/ml<br>pTau: 97pg/ml<br>Tau: 671pg/ml<br>2. FDG-PET: cortical hypometabolism in both frontomesial areas (accentuated on the right side), in both precuneus regions. and cerebellar<br>3. Amyloid-PET: cortical amyloid load**	**negative<br>Goldman Score: 4**
**EOD-19**	**m**	**AD**	**51**	**1. episodic memory<br>2. word retrieval<br>3. seizures**	**1.no pathological findings**	**1. CSF:<br>Aβ: 714pg/ml<br>pTau: 79pg/ml<br>Tau: 638 pg/ml<br>2. FDG-PET: hypometabolism in both temporal and parietal lobes and in the left precuneus region<br>3. Amyloid-PET: cortical amyloid load (emphasis in the left precuneus region)**	**1. mother: AD (AAO 53)<br>2. Brother: AD (AAO 47, APP duplication)<br>3. two maternal aunts: AD (AAO 50)<br>Goldman Score: 1**

No.	Sex	Diagnosis (other relevant diagnoses)	AAO (y)	Symptoms	MRI	Other biomarkers	Family History (Goldman Score)
**EOD-19(2)**	**m**	**AD**	**47**	**1. episodic memory**	**1.no pathological findings**	**1. CSF:<br>Aβ: 598pg/ml<br>pTau: 99pg/ml<br>Tau: 663pg/ml<br>2. FDG-PET: hypometabolism in the left temporal lobe<br>3. Amyloid-PET: cortical amyloid load**	**1. mother: AD (AAO 53)<br>2. Brother: AD (AAO 51, APP duplication)<br>3. maternal aunt: AD (AAO 50)<br>Goldman Score: 1**
EOD-20	m	AD	57	1. episodic memory<br>2. seizures	1. global atrophy, especially hippocampal atrophy bilateral<br>2. moderate white matter hyperintensities	1. CSF:<br>Aβ: 176pg/ml<br>pTau: 34pg/ml<br>Tau: 535pg/ml<br>2. FDG-PET: hypometabolism in both frontal, parietal and temporal lobes as well as basal ganglia and thalamus region<br>3. Amyloid-PET: cortical amyloid load in frontal, parietal, occipital and temporal lobes (accentuated in frontal lobes and left temporal lobe) and in both precuneus regions (accentuated on the left) and basal ganglia (accentuated on the left)	unknown<br>Goldman Score: 4.5
**EOD-21**	**m**	**CAA**	**54**	**1. episodic memory**	**1. no significant atrophy<br>2. moderate white matter hyperintensities<br>3. numerous cortical supratentorial microhemorrhage (accentuated in the right insula, left occipital and temporal lobe)**	**1. CSF:<br>Aβ: 185pg/ml<br>pTau: 88pg/ml<br>Tau: 1342pg/ml<br>2. FDG-PET: normal findings<br>3. Amyloid-PET: cortical amyloid load, accentuated in frontobasal, parietal and temporolateral areas**	**Mother: unspecified dementia (AAO unknown)<br>Goldman Score: 3.5**

No.	Sex	Diagnosis (other relevant diagnoses)	AAO (y)	Symptoms	MRI	Other biomarkers	Family History (Goldman Score)
EOD-22	m	AD	49	1. episodic memory	1.no pathological findings	1. CSF:<br>Aβ: 242pg/ml<br>pTau:<br>101pg/ml<br>Tau: 489pg/ml<br>2. FDG-PET: hypometabolism in both parietal lobes, accentuated on<br>3. Amyloid-PET: cortical amyloid load, accentuated in both frontal lobes and basal ganglia	negative<br>Goldman score: 4
EOD-23	f	AD (Multiple Sclerosis)	36	1. episodic memory<br>2. spatial orientation	1. diffuse subcortical atrophy, accentuated on the temporal lobe<br>2. demyelinating supratentorial lesions, Gd-enhanced lesions of the left superior frontal gyrus	1. CSF:<br>Aβ: 465pg/ml<br>pTau: 115pg/ml<br>Tau: 1003pg/ml<br>OCB positive<br>2. FDG-PET: mild hypometabolism in the left precuneus region<br>3. Amyloid-PET: cortical amyloid load accentuated in the left precuneus region	1. Mother: psychiatric disease vs. dementia (died at an early age)<br>2. maternal grandfather: unspecified dementia (AAO unknown)<br>3. maternal uncle: unspecified dementia (AAO unknown)<br>Goldman score: 1
EOD-24	m	AD	53	1. episodic memory<br>2. spatial orientation	1.mild global atrophy	1. CSF:<br>Aβ: 548pg/ml<br>pTau: 84pg/ml<br>Tau: 595pg/ml<br>2. FDG-PET: hypometabolism in both parietal lobes > both frontal and temporal lobes, caudate nucleus and thalamus<br>3. Amyloid-PET: cortical amyloid load	1.mother: unspecified dementia (AAO unknown, died at the age of 80)<br>Goldman score: 3.5
EOD-25	f	AD	51	1. episodic memory<br>2. aphasia	1. global atrophy with temporal and hippocampal emphasis<br>2. moderate white matter hyperintensities	1. CSF: missing<br>2. FG-PET: missing<br>3. Amyloid-PET: missing	1.father: AD (AAO 72)<br>Goldman score: 3.5

No.	Sex	Diagnosis (other relevant diagnoses)	AAO (y)	Symptoms	MRI	Other biomarkers	Family History (Goldman Score)
EOD-26	f	AD	56	1. episodic memory	1. mild biparietal atrophy<br>2. mild white matter hyperintensities	1. CSF:<br>Aβ: 185pg/ml<br>pTau:<br>59pg/ml<br>Tau: 374pg/ml<br>2. FDG-PET: hypometabolism in the left temporolateral area and adjacent parietal lobe<br>3. Amyloid-PET: cortical amyloid load accentuated on both frontal and temporal lobes, precuneus and caudate nucleus regions	negative<br>Goldman score: 4
EOD-27	f	AD	57	1. episodic memory	1. mild frontoparietal atrophy<br>2. moderate white matter hyperintensities	1. CSF:<br>Aβ: 673pg/ml<br>pTau: 143pg/ml<br>Tau: 1682pg/ml<br>2. FDG-PET: hypometabolism in the left parietotemporal area<br>3. Amyloid-PET: cortical amyloid load	negative<br>Goldman score: 4
EOD-28	m	AD	54	1. episodic memory<br>2. hyposmia<br>3. dyscalculia	1.no pathological findings	1. CSF:<br>Aβ: 310pg/ml<br>pTau: 55pg/ml<br>Tau: 483pg/ml<br>2. FDG-PET: cortical hypometabolism in the right frontal and both parietotemporal and precuneus regions<br>3. Amyloid-PET: cortical amyloid load accentuated on the frontal more than on the occipital lobes and basal ganglia	negative<br>Goldman score: 4

No.	Sex	Diagnosis (other relevant diagnoses)	AAO (y)	Symptoms	MRI	Other biomarkers	Family History (Goldman Score)
EOD-29	m	AD	54	1. episodic memory<br>2. behavioural symptoms (aggressive behaviour, apathy, loss of empathy)	1. atrophy of the parietal and occipital lobe (bilateral), cerebellar atrophy	1. CSF:<br>Aβ: 380pg/ml<br>pTau: 95pg/ml<br>Tau: 776pg/ml<br>2. FDG-PET: hypometabolism in parietal lobes (left>right) reaching the left postcentral and mid-temporal areas, hypometabolism in both occipitolateral and precuneus regions<br>3. Amyloid-PET: missing	negative<br>Goldman score:4
EOD-30	m	AD	64	1. episodic memory<br>2. concentration	1. no atrophy<br>2. mild white matter hyperintensities	1. CSF:<br>Aβ: 203pg/ml<br>pTau: 130pg/ml<br>Tau: 440pg/ml<br>2. FDG-PET: hypometabolism in parietal and temporal lobes (accentuated on the right side) and in the right precuneus region<br>3. Amyloid-PET: inhomogeneous cortical amyloid load in frontoparietotemporal and precuneus regions.	negative<br>Goldman score: 4
EOD-31	m	mixed dementia (AD+VD)	58	1. episodic memory<br>2. word retrieval	1. supratentorial atrophy<br>2. chronic peritrigonal infarction on both sides and severe white matter hyperintensities	1. CSF:<br>Aβ: reduced (no exact levels available)<br>Tau/pTau: elevated (no exact levels available)<br>2. FDG-PET: missing<br>3. Amyloid-PET: pathological amyloid load frontotemporal	1.Mother: unspecified dementia (AAO 70)<br>2.grandmother: unspecified dementia (AAO 78)<br>Goldman score: 3.5

No.	Sex	Diagnosis (other relevant diagnoses)	AAO (y)	Symptoms	MRI	Other biomarkers	Family History (Goldman Score)
EOD-32	m	FTD/svPPA	61	1. episodic memory<br>2. word retrieval<br>3. naming of objects	1.significant atrophy hippocampal, temporal and in the insular cortex	1. CSF:<br>Aβ: 978pg/ml<br>pTau: 57pg/ml<br>Tau: 565pg/ml<br>2. FDG-PET: significant hypometabolism in both temporal lobes, mild hypometabolism in both frontal lobes<br>3. Amyloid-PET: normal findings	negative<br>Goldman score: 4
EOD-33	f	AD	62	1. episodic memory<br>2. word retrieval	1. moderate supratentorial atrophy<br>2. mild white matter hyperintensities	1. CSF: missing<br>2. FDG-PET: missing<br>3. Amyloid-PET: missing	unknown<br>Goldman score: 4.5
EOD-34	f	AD	59	1. episodic memory<br>2. spatial orientation	1. diffuse global atrophy	1. CSF: missing<br>2. FDG-PET: missing<br>3. Amyloid-PET: cortical amyloid load frontoparietotemporal	1.Mother: unspecified dementia (AAO>80)<br>2.Maternal grandmother and grandaunt: unspecified dementia (late onset)<br>Goldman score: 2
EOD-35	m	AD	55	1. episodic memory	1.moderate global atrophy with no dementia-specific pattern	1. CSF:<br>Aβ: 397pg/ml<br>pTau: 58pg/ml<br>Tau: 563pg/ml<br>2. FDG-PET: missing<br>3 Amyloid-PET: cortical amyloid load	1.Mother: vascular dementia (AAO >70)<br>Goldman score: 3.5
EOD-36	m	AD	64	1. episodic memory<br>2. spatial orientation	1.supratentorial atrophy accentuated on the parietal and temporal lobe (including hippocampus)	1. CSF:<br>Aβ: 512pg/ml<br>pTau: 161pg/ml<br>Tau: 1627pg/ml<br>2. FDG-PET: hypometabolism in both temporal and parietal lobes<br>3. Amyloid-PET: cortical amyloid load	1.Brother: unspecified dementia (AAO 62)<br>2.father: unspecified dementia (AAO 80)<br>Goldman score:2

No.	Sex	Diagnosis (other relevant diagnoses)	AAO (y)	Symptoms	MRI	Other biomarkers	Family History (Goldman Score)
EOD-37	f	AD	52	1. episodic memory	1.significant supratentorial atrophy	1. CSF:<br>Aβ: 162pg/ml<br>pTau: 23pg/ml<br>Tau: 170pg/ml<br>2. FDG-PET: hypometabolism in both frontal, parietal and temporal lobes, in lateral occipital cortex and both precuneus regions<br>3. Amyloid-PET: cortical amyloid, in both precuneus regions and in basal ganglia regions	1.mother: unspecified dementia (AAO 80)<br>Goldman score: 3.5
EOD-38	f	AD	52	1. episodic memory<br>2. word retrieval<br>3. concentration	1.generalized supratentorial atrophy, accentuated on the frontal and temporal lobe<br>2.mild white matter hyperintensities	1. CSF:<br>Aβ: 139pg/ml<br>pTau: 65pg/ml<br>Tau: 515pg/ml<br>2. FDG-PET: hypometabolism in both parietal, temporal and frontal lobes as well as in both precuneus regions<br>3. Amyloid-PET: cortical amyloid load in both parietal, temporal and frontal lobes and in both precuneus regions	1.father: unspecified dementia (AAO>65)<br>Goldman score: 3.5
EOD-39	f	AD	63	1. episodic memory	1.no pathological findings	1. CSF:<br>Aβ: 366pg/ml<br>pTau: 46pg/ml<br>Tau: 197pg/ml<br>2. FDG-PET: normal findings<br>3. Amyloid-PET: amyloid load in both frontal lobes and in the left precuneus and basal ganglia regions	1.mother: AD (AAO 70)<br>2.maternal aunt: AD (AAO 60)<br>Goldman score: 3
EOD-40	f	AD	55	1. episodic memory<br>2. dyscalculia<br>3. spatial orientation	1. no atrophy<br>2.mild white matter hyperintensities	1. CSF:<br>Aβ: 350pg/ml<br>pTau: 65pg/ml<br>Tau: 490pg/ml<br>2. FDG-PET: hypometabolism in both precuneus regions and in both frontal, temporal and parietal lobes<br>3. Amyloid-PET: cortical amyloid load	negative<br>Goldman score: 4

No.	Sex	Diagnosis (other relevant diagnoses)	AAO (y)	Symptoms	MRI	Other biomarkers	Family History (Goldman Score)
EOD-41	m	AD	58	1. episodic memory<br>2. spatial orientation	1. mild global atrophy accentuated on the parietal and occipital lobe including precuneus regions<br>2.mild white matter hyperintensities	1. CSF: missing<br>2. FDG-PET: hypometabolism parietotemporal and in the left frontal and occipital lobe<br>3. Amyloid-PET: cortical amyloid load accentuated on both frontal lobes	1. mother: unspecified dementia (AAO >80)<br>2. Maternal grandmother: unspecified dementia (AAO >80)<br>Goldman score: 3.5
EOD-42	m	AD	39	1. episodic memory	1. no atrophy<br>2. no other pathological findings	1. CSF: Aβ: 452pg/ml<br>pTau:<br>38pg/ml<br>Tau: 191pg/ml<br>2. FDG-PET: cortical hypometabolism in both temporomesial areas<br>3. Amyloid-PET: inhomogeneous cortical amyloid load	negative<br>Goldman score: 4
EOD-43	m	AD	63	1. episodic memory<br>2. concentration	1. mild global atrophy<br>2. mild white matter hyperintensities	1. CSF:<br>Aβ: 185pg/ml<br>pTau: 42pg/ml<br>Tau: 258pg/ml<br>2. FDG-PET: hypometabolism in the left parietooccipital lobe<br>3. Amyloid-PET: cortical amyloid load in frontal and parietooccipital lobes and precuneus regions	negative<br>Goldman score: 4
EOD-44	f	AD/lpPPA	58	1. word retrieval<br>2. episodic memory	1. mild global atrophy<br>2. moderate white matter hyperintensities	1. CSF:<br>Aβ: 318pg/ml<br>pTau: 93pg/ml<br>Tau: 905pg/ml<br>2. FDG-PET: missing<br>3. Amyloid-PET: missing	1.Mother: oncological disease associated with cognitive dysfunction (AAO >70)<br>2.father: alcohol abuse associated with cognitive dysfunction (AAO >60)<br><br>Goldman score: 3.5
EOD-45	m	AD	65	1. episodic memory<br>2. spatial orientation<br>3. gait disturbance	1. hippocampal atrophy<br>2. AVM in the left occipital lobe and left cerebellum	1. CSF: missing<br>2. FDG-PET: hypometabolism in both frontal, parietal and temporal lobes as well as precuneus regions<br>3. Amyloid-PET: cortical amyloid load	negative<br>Goldman score: 4

No.	Sex	Diagnosis (other relevant diagnoses)	AAO (y)	Symptoms	MRI	Other biomarkers	Family History (Goldman Score)
EOD-46	f	CBS+ AD	51	1. visuoconstructive deficits<br>2. psychomotor slowing<br>3. spatial orientation<br>4. word retrieval<br>5. apraxia<br>6. left hemispatial neglect<br>7. coordination<br>8. depression	1. mild global atrophy accentuated on the occipital and parietal lobe bilateral<br>2.mild to moderate white matter hyperintensities	1. CSF:<br>Aβ: 197pg/ml<br>pTau: 33pg/ml<br>Tau: 230pg/ml<br>2. FDG-PET: hypometabolism parietotemporal, occipitolateral and frontolateral (both sides)<br>3. Amyloid-PET: cortical amyloid load in both frontal lobes and to a lesser extent in both parietal, occipital and temporal lobes<br>4. DaT-SPECT: reduced uptake in both putamina (accentuated on the left)	1.father: AD (AAO>80)<br>Goldman score: 3.5
EOD-47	f	AD	54	1. episodic memory<br>2. spatial orientation<br>3. word retrieval	1. No atrophy<br>2.mild white matter hyperintensities	1. CSF:<br>Aβ: 308pg/ml<br>pTau: 70pg/ml<br>Tau: 791pg/ml<br>2. FDG-PET: normal findings<br>3. Amyloid-PET: global inhomogeneous cortical amyloid load	negative<br>Goldman score: 4
EOD-48	m	bvFTD	57	1. behavioural symptoms (disinhibition, loss of empathy)	1. no atrophy<br>2.mild white matter hyperintensities	1. CSF:<br>Aβ: 1023pg/ml<br>pTau: 45pg/ml<br>Tau: 482pg/ml<br>2. FDG-PET: significant hypometabolism from the right temporal to parietal and frontal lobes, to a lesser extent in left temporal and frontal lobes<br>3. Amyloid-PET: normal findings	negative<br><br>Goldman score: 4

No.	Sex	Diagnosis (other relevant diagnoses)	AAO (y)	Symptoms	MRI	Other biomarkers	Family History (Goldman Score)
EOD-49	m	FTD/nfPPA +ALS	58	1. word retrieval<br>2. dysarthria<br>3. behavioural symptoms (emotional imbalance)<br>4. gait disturbance<br>5. episodic memory	1.no pathological findings	1. CSF:<br>Aβ: 539pg/ml<br>pTau: 42pg/ml<br>Tau: 298pg/ml<br>2 FDG-PET: significant hypometabolism in both frontotemporal lobes (accentuated on the right), insular cortex and parietal lobes<br>3. Amyloid-PET: normal findings	negative<br>Goldman score: 4
**EOD-50**	**f**	**FTD (bvFTD+nf PPA)**	**55**	**1. word-retrieval**<br>**2. behavioural symptoms (aggressive behaviour and emotional instability)**<br>**3. increased appetite**	**1. generalized supratentorial atrophy accentuated on the left hemisphere (frontal, parietal and temporal lobe as well as insular cortex), to a lesser extent frontoparietal atrophy on the right side; Hippocampal atrophy accentuated on the left side**<br>**2. mild white matter hyperintensities**	**1. CSF:**<br>**Aβ: 711pg/ml**<br>**pTau: 34pg/ml**<br>**Tau: 520pg/ml**<br>**2. FDG-PET: hypometabolism accentuated on the left hemisphere (reaching from the frontal lobe to the insular cortex, parietal and anterior temporal lobe), hypometabolism in the left precuneus region and left rostral caudate nucleus**<br>**3. Amyloid-PET:missing**	**1.mother: FTD/nfPPA (AAO>70)**<br>**Goldman score: 3.5**
EOD-51	f	FTD/svPPA	62	1. word-retrieval<br>2. executive functioning	1. no atrophy<br>2. mild white matter hyperintensities	1. CSF:<br>Aβ: 516pg/ml<br>pTau: 32pg/ml<br>Tau: 232pg/ml<br>2. FDG-PET: hypometabolism accentuated on the left frontolateral lobe and insular cortex<br>3. Amyloid-PET: normal findings	negative<br>Goldman score: 4

No.	Sex	Diagnosis (other relevant diagnoses)	AAO (y)	Symptoms	MRI	Other biomarkers	Family History (Goldman Score)
EOD-52	m	AD	57	1. episodic memory<br>2. word-retrieval	1. mild temporoparietal and hippocampal atrophy accentuated on the left side	1. CSF:<br>Aβ: 259pg/ml<br>pTau: 49pg/ml<br>Tau: 331pg/ml<br>2. FDG-PET: cortical hypometabolism temporoparietal (accentuated on the left)<br>3. Amyloid-PET: cortical amyloid load	negative<br>Goldman score: 4
EOD-53	m	AD	57	1. episodic memory	1. moderate global atrophy accentuated on the right side, mild cerebellar atrophy<br>2. mild white matter hyperintensities	1. CSF: missing<br>2. FDG-PET: inhomogeneous cortical hypometabolism in the right parietal and temporal lobes and precuneus region<br>3. Amyloid-PET: global cortical tracer uptake accentuated in both frontal lobes and in the left temporoparietal lobe	negative<br>Goldman score: 4
EOD-54	m	AD	59	1. episodic memory<br>2. spatial orientation	1. severe global atrophy with hippocampal accentuation<br>2. mild white matter hyperintensities	1. CSF: missing<br>2. FDG-PET: missing<br>3. Amyloid-PET: missing	1. mother: (unspecified) dementia (AAO 58)<br>2. sister: (unspecified) dementia (AAO 65)<br>Goldman score: 1
EOD-55	m	AD	49	1. episodic memory<br>2. spatial orientation	1. moderate global atrophy accentuated on both parietal lobes<br>2. mild white matter hyperintensities<br>3. small cavernoma in the left occipital lobe	1. CSF:<br>Aβ: 441pg/ml<br>pTau: 86pg/ml<br>Tau: 435pg/ml<br>2. FDG-PET: hypometabolism in both parietal lobes and precuneus regions (accentuated on the left)<br>3. Amyloid-PET: inhomogeneous cortical amyloid load accentuated in both frontobasal lobes and the left temporal lobe	negative<br>Goldman score: 4

No.	Sex	Diagnosis (other relevant diagnoses)	AAO (y)	Symptoms	MRI	Other biomarkers	Family History (Goldman Score)
EOD-56	m	AD	61	1. episodic memory<br>2. spatial orientation	1. no atrophy<br>2. moderate white matter hyperintensities	1. CSF:<br>Aβ: 250pg/ml<br>pTau: 28pg/ml<br>Tau: 162pg/ml<br>2. FDG-PET: cortical hypometabolism in both frontal and temporal lobes (accentuated on the right)<br>3. Amyloid-PET: cortical amyloid load	1. mother: (unspecified) dementia (AAO 80)<br>Goldman score: 3.5
EOD-57	f	AD/lpPPA	57	1. word-retrieval	1. mild atrophy in both frontal and temporal lobes<br>2. symmetric widened sylvian fissure	1. CSF:<br>Aβ: 246pg/ml<br>pTau: 68pg/ml<br>Tau: 473pg/ml<br>2. FDG-PET: inhomogeneous hypometabolism accentuated on the left temporal, parietal and occipital lobes<br>3. Amyloid-PET: inhomogeneous cortical amyloid load	negative<br>Goldman score: 4
EOD-58	f	mixed dementia (AD +VD)	64	1. word-retrieval<br>2. episodic memory<br>3. dyscalculia	1. no atrophy<br>2. moderate white matter hyperintensities	1. CSF:<br>Aβ: 335pg/ml<br>pTau: 89pg/ml<br>Tau: 718pg/ml<br>2. FDG-PET: cortical hypometabolism in both parietal and temporal lobes<br>3. Amyloid-PET: normal findings	1. grandmother: (unspecified) dementia (AAO <52)<br>2. aunt: (unspecified) dementia (AAO <75)<br>Goldman score: 3
EOD-59	m	bvFTD	52	1. behavioural symptoms (aggressive behaviour, apathy)<br>2. acoustic hallucinations	1. no atrophy<br>2. mild white matter hyperintensities	1. CSF:<br>Aβ: 299pg/ml<br>pTau: 24pg/ml<br>Tau: <135pg/ml<br>2. FDG-PET: hypometabolism in both frontal lobes (accentuated on the left), the left insula and temporal lobe<br>3. Amyloid-PET: normal findings<br>4. DaT-SPECT: reduced uptake in the left caudate nucleus and putamen	negative<br>Goldman score:4

No.	Sex	Diagnosis (other relevant diagnoses)	AAO (y)	Symptoms	MRI	Other biomarkers	Family History (Goldman Score)
EOD-60	f	AD	49	1. episodic memory<br>2. spatial orientation	1. no atrophy	1. CSF:<br>Aβ: 376pg/ml<br>pTau: 193pg/ml<br>Tau: 2074pg/ml<br>2. FDG-PET: hypometabolism in the left temporolateral lobe and precuneus region, slight hypometabolism in the left frontal and parietal lobe<br>3. Amyloid-PET: cortical amyloid load	1. mother: (unspecified) dementia (AAO 50)<br>Goldman score: 3

AAO, age at onset; Aβ, Amyloid beta; AD, Alzheimer's disease; ALS, amyotrophic lateral sclerosis; CAA, Cerebral Amyloid Angiopathy; CBS, Corticobasal syndrome; CSF, cerebrospinal fluid; DaT-SPECT, Dopamine transporter single-photon emission computed tomography; DVA, developmental venous anomaly; FTD, frontotemporal dementia (bvFTD, behavioural frontotemporal dementia); FDG-PET, 18-fluorodeoxyglucose (FDG) positron emission tomography (PET); MCI, mild cognitive impairment; PPA, primary progressive aphasia (lpPPA, logopenic primary progressive aphasia; nfPPA, non-fluent primary progressive aphasia); PD, Parkinson's Disease; pTau, phosphorylated Tau; Tau, total Tau; VD, vascular dementia

Modified Goldman score (family history), *Goldman, Farmer et al. 2005: (*1) autosomal dominant - three affected individuals over two generations with one person being a first-degree relative to the others; (2) familial aggregation - three relatives affected without fulfilling the criteria for autosomal dominant inheritance; (3) single affected first-degree relative with an age at onset under 65 years; (3.5) single affected first-degree relative with an age at onset over 65 years; (4) negative family history; (4.5) unknown family history

Additional file 1: Table S2. Gene list for alignment with rare variants in the cohort

	Gene	Reference	PMID
A	AARD	Zhang (2019)	30503768
	ABCA1	Beecham (2018)	30569016
	ABCA7	Vardarajan (2018)	29688227
	ABCC6	Raghavan (2018)	30009200
	ABCD4	Patel (2019)	30924900
	ABI3	Conway (2018)	30326945
	ABI3BP	Raghavan (2018)	30009200
	AC099552	Ma (2019)	31180460
	ACAD10	Raghavan (2018)	30009200
	ACE	Marioni (2018)	29777097
	ACOX2	Raghavan (2018)	30009200
	ADAM10	Marioni (2018)	29777097
	ADAM18	Raghavan (2018)	30009200
	ADAMTS1	Kunkle (2019)	30820047
	ADAMTS4	Marioni (2018)	29777097
	ADM5	Raghavan (2018)	30009200
	AHNAK	Vardarajan (2018)	29688227
	AKAP9	Vardarajan (2018)	29688227
	AKNAD1	Zhang (2019)	30503768
	AKR1C2	Raghavan (2018)	30009200
	ALDH16A1	Raghavan (2018)	30009200
	ALG1	Raghavan (2018)	30009200
	ALPL	Patel (2019)	30924900
	ALS2	Cirulli (2015)	25700176
	AMH	Raghavan (2018)	30009200
	ANG	Cirulli (2015)	25700176
	ANGPTL3	Patel (2019)	30924900
	ANGPTL7	Raghavan (2018)	30009200
	ANK3	Raghavan (2018)	30009200
	ANKRD27	Raghavan (2018)	30009200
	ANKRD53	Raghavan (2018)	30009200
	ANO7	Raghavan (2018)	30009200
	ANXA5	Zhang (2019)	30503768
	APLP1	Raghavan (2018)	30009200
	APOB	Raghavan (2018)	30009200
	APOE	Saunders (1993)	8350998
	APP	Goate (1991)	1671712
	ARAP2	Raghavan (2018)	30009200
	ARHGAP1	Raghavan (2018)	30009200
	ARHGAP31	Raghavan (2018)	30009200
	ARHGEF11	Raghavan (2018)	30009200
	ARPC1B	Raghavan (2018)	30009200
	ART3	Raghavan (2018)	30009200
	ASB13	Zhang (2019)	30503768
	ASCC1	Zhang (2019)	30503768
	ASNA1	Raghavan (2018)	30009200
	ASRGL1	Vardarajan (2018)	29688227
	ASXL3	Raghavan (2018)	30009200
	ATG4D	Raghavan (2018)	30009200
	ATXN1	Bertram (2008)	18976728
	ATXN2	Cirulli (2015)	25700176
B	B4GALNT4	Raghavan (2018)	30009200
	BACE1	Li (2019)	31570097
	BANK1	Raghavan (2018)	30009200
	BCAN	Raghavan (2018)	30009200
	BCKDK	Raghavan (2018)	30009200
	BCL2L11	Patel (2019)	30924900
	BEND3	Raghavan (2018)	30009200
	BEND4	Raghavan (2018)	30009200
	BICD2	Raghavan (2018)	30009200
	BID	Raghavan (2018)	30009200
	BIN1	Hollingworth (2011)	21460840
	BLOC1S3	Raghavan (2018)	30009200
	BOLL	Couthouis (2011)	22065782
	BRCA2	Raghavan (2018)	30009200
	BRD4	Raghavan (2018)	30009200
	BUD13	Raghavan (2018)	30009200
	BZRAP1	Marioni (2018)	29777097
C	C10orf131	Raghavan (2018)	30009200
	C11orf40	Raghavan (2018)	30009200
	C14orf28	Patel (2019)	30924900
	C17orf78	Raghavan (2018)	30009200
	C19orf57	Raghavan (2018)	30009200
	C1orf173	Zhang (2019)	30503768
	C22orf39	Raghavan (2018)	30009200
	C5	Raghavan (2018)	30009200
	C9orf72	Cirulli (2015)	25700176
	CAMKMT	Raghavan (2018)	30009200
	CAPZA3	Beecham (2018)	30569016
	CARF	Raghavan (2018)	30009200
	CASP10	Raghavan (2018)	30009200
	CASP7	Zhang (2019)	30503768
	CASS4	Lambert (2013)	24162737
	CATSPERG	Raghavan (2018)	30009200
	CCDC122	Raghavan (2018)	30009200
	CCDC129	Vardarajan (2018)	29688227
	CCDC178	Raghavan (2018)	30009200
	CCDC18	Patel (2019)	30924900
	CCDC28B	Raghavan (2018)	30009200
	CCNF	Williams (2016)	27080313
	CCNYL1	Beecham (2018)	30569016
	CD2AP	Hollingworth (2011)	21460840

	Gene	Reference	PMID
	CD300C	Raghavan (2018)	30009200
	CD33	Hollingworth (2011)	21460840
	CD84	Raghavan (2018)	30009200
	CDCA7L	Raghavan (2018)	30009200
	CDCP2	Raghavan (2018)	30009200
	CDH8	Beecham (2018)	30569016
	CELF1	Lambert (2013)	24162737
	CELSR1	Patel (2019)	30924900
	CENPQ	Raghavan (2018)	30009200
	CEP290	Patel (2019)	30924900
	CEP72	Raghavan (2018)	30009200
	CES3	Raghavan (2018)	30009200
	CHCHD10	Cirulli (2015)	25700176
	CHMP2B	Cirulli (2015)	25700176
	CHRNA3	Kuźma (2018)	29865062
	CHRNA4	Kuźma (2018)	29865062
	CHRNB4	Kuźma (2018)	29865062
	CHTF18	Raghavan (2018)	30009200
	CIC	Raghavan (2018)	30009200
	CLCN1	Raghavan (2018)	30009200
	CLDN7	Patel (2019)	30924900
	CLIC5	Raghavan (2018)	30009200
	CLU	Harold (2009)	19734902
	COBL	Patel (2019)	30924900
	COG4	Beecham (2018)	30569016
	COL15A1	Raghavan (2018)	30009200
	COQ4	Raghavan (2018)	30009200
	CORO2B	Raghavan (2018)	30009200
	CPA1	Raghavan (2018)	30009200
	CPSF6	Couthouis (2011)	22065782
	CPVL	Vardarajan (2018)	29688227
	CR1	Vardarajan (2018)	29688227
	CRBN	Raghavan (2018)	30009200
	CRYZ	Raghavan (2018)	30009200
	CSF1R	Sassi (2018)	29544907
	CTSC	Ferrari (2014)	24943344
	CUBN	Patel (2019)	30924900
	CUGBP2	Wijsman (2011)	21379329
	CX3CR1	Dworzak (2015)	26038823
	CYB561A3	Vardarajan (2018)	29688227
	CYB5R4	Raghavan (2018)	30009200
**D**	DAAM2	Raghavan (2018)	30009200
	DAO	Cirulli (2015)	25700176
	DAZ1	Couthouis (2011)	22065782
	DAZ2	Couthouis (2011)	22065782
	DAZ3	Couthouis (2011)	22065782

	Gene	Reference	PMID
	DAZAP1	Couthouis (2011)	22065782
	DCTN1	Cirulli (2015)	25700176
	DEF6	Raghavan (2018)	30009200
	DEFB125	Raghavan (2018)	30009200
	DENND4A	Raghavan (2018)	30009200
	DHRS11	Raghavan (2018)	30009200
	DLEC1	Patel (2019)	30924900
	DNAH14	Patel (2019)	30924900
	DNAJC10	Raghavan (2018)	30009200
	DNASE1	Raghavan (2018)	30009200
	DNM1	Raghavan (2018)	30009200
	DOC2A	Raghavan (2018)	30009200
	DOCK4	Patel (2019)	30924900
	DOK5	Raghavan (2018)	30009200
	DPP6	Cacace (2019)	30874922
	DSG2	Lambert (2013)	24162737
	DTNA	Raghavan (2018)	30009200
	DTYMK	Zhang (2019)	30503768
	DUSP19	Raghavan (2018)	30009200
	DVL2	Raghavan (2018)	30009200
	DYSF	Raghavan (2018)	30009200
**E**	ECM2	Raghavan (2018)	30009200
	ECSIT	Raghavan (2018)	30009200
	EGFL7	Raghavan (2018)	30009200
	EIF2A	Raghavan (2018)	30009200
	EIF3K	Raghavan (2018)	30009200
	EIF3L	Raghavan (2018)	30009200
	ELAVL1	Couthouis (2011)	22065782
	ELAVL2	Couthouis (2011)	22065782
	ELAVL3	Couthouis (2011)	22065782
	ELAVL4	Couthouis (2011)	22065782
	ELL	Raghavan (2018)	30009200
	ELP3	Cirulli (2015)	25700176
	ENO3	Raghavan (2018)	30009200
	ENOX1	Couthouis (2011)	22065782
	EPHA1	Vardarajan (2018)	29688227
	EPHA4	Shen (2010)	20100581
	ERBB4	Woo (2011)	21829755
	ERLIN2	Teranishi (2012)	22771797
	EWSR1	Couthouis (2011)	22065782
	EXOSC5	Raghavan (2018)	30009200
**F**	F2RL1	Raghavan (2018)	30009200
	FAM124A	Raghavan (2018)	30009200
	FAM134A	Raghavan (2018)	30009200
	FAM171A2	Patel (2019)	30924900
	FAM184A	Raghavan (2018)	30009200

	Gene	Reference	PMID
	FAM188B	Vardarajan (2018)	29688227
	FAM208A	Raghavan (2018)	30009200
	FAM217A	Raghavan (2018)	30009200
	FAM47E	Raghavan (2018)	30009200
	FARP2	Raghavan (2018)	30009200
	FBP1	Raghavan (2018)	30009200
	FERMT2	Lambert (2013)	24162737
	FFAR1	Raghavan (2018)	30009200
	FIG4	Cirulli (2015)	25700176
	FISP2	Beecham (2018)	30569016
	FN1	Raghavan (2018)	30009200
	FOXG1	Ma (2019)	31180460
	FOXK1	Raghavan (2018)	30009200
	FOXRED1	Raghavan (2018)	30009200
	FRMPD2	Raghavan (2018)	30009200
	FSCN3	Raghavan (2018)	30009200
	FUS	Couthouis (2011)	22065782
	FUT5	Raghavan (2018)	30009200
**G**	G3BP1	Couthouis (2011)	22065782
	GABBR2	Raghavan (2018)	30009200
	GALR3	Raghavan (2018)	30009200
	GANAB	Vardarajan (2018)	29688227
	GAS2L3	Raghavan (2018)	30009200
	GAS6	Raghavan (2018)	30009200
	GCDH	Raghavan (2018)	30009200
	GCFC2	Raghavan (2018)	30009200
	GIMAP2	Patel (2019)	30924900
	GJB7	Raghavan (2018)	30009200
	GK2	Raghavan (2018)	30009200
	GLE1	Cirulli (2015)	25700176
	GLIS3	Patel (2019)	30924900
	GPAA1	Ma (2019)	31180460
	GPATCH1	Raghavan (2018)	30009200
	GPR63	Raghavan (2018)	30009200
	GRID2IP	Raghavan (2018)	30009200
	GTSE1	Patel (2019)	30924900
**H**	HACL1	Raghavan (2018)	30009200
	HCCAT5	Beecham (2018)	30569016
	HDGFRP2	Raghavan (2018)	30009200
	HELLS	Raghavan (2018)	30009200
	HELZ2	Patel (2019)	30924900
	HERC3	Raghavan (2018)	30009200
	HFE	Sampietro (2001)	11445256
	HID1	Raghavan (2018)	30009200
	HLA-B	Raghavan (2018)	30009200
	HLA-DOA	Raghavan (2018)	30009200
	HLA-DQA1	Raghavan (2018)	30009200
	HLA-DRB1	Lambert (2013)	24162737
	HLA-DRB5	Lambert (2013)	24162737
	HMOX1	Raghavan (2018)	30009200
	HNRNPA0	Couthouis (2011)	22065782
	HNRNPA1	Couthouis (2011)	22065782
	HNRNPA2B1	Couthouis (2011)	22065782
	HNRNPM	Couthouis (2011)	22065782
	HOXC11	Raghavan (2018)	30009200
	HTR3A	Zhang (2019)	30503768
	HTR3E	Raghavan (2018)	30009200
**I**	ICAM3	Raghavan (2018)	30009200
	IFT140	Raghavan (2018)	30009200
	IGF2BP2	Couthouis (2011)	22065782
	IGF2BP3	Couthouis (2011)	22065782
	IGHJ6	Zhang (2019)	30503768
	IGHV3	Ma (2019)	31180460
	IGLL5	Raghavan (2018)	30009200
	INPP5D	Patel (2019)	30924900
	IQCK	Kunkle (2019)	30820047
	IQGAP1	Raghavan (2018)	30009200
	ISYNA1	Ma (2019)	31180460
	ITGA8	Raghavan (2018)	30009200
	IVNS1ABP	Raghavan (2018)	30009200
**J**	JAG2	Raghavan (2018)	30009200
	JMJD4	Zhang (2019)	30503768
	JPH2	Raghavan (2018)	30009200
**K**	KANSL1	Patel (2019)	30924900
	KANSL1L	Raghavan (2018)	30009200
	KANSL3	Zhang (2019)	30503768
	KBTBD12	Raghavan (2018)	30009200
	KCNK13	Zhang (2019)	30503768
	KCTD13	Raghavan (2018)	30009200
	KCTD21	Raghavan (2018)	30009200
	KHK	Raghavan (2018)	30009200
	KIAA0141	Raghavan (2018)	30009200
	KIF19	Patel (2019)	30924900
	KIF24	Raghavan (2018)	30009200
	KMT2E	Raghavan (2018)	30009200
	KPRP	Raghavan (2018)	30009200
	KRT15	Raghavan (2018)	30009200
	KRT86	Raghavan (2018)	30009200
	KRTAP4-3	Raghavan (2018)	30009200
**L**	L3MBTL1	Raghavan (2018)	30009200
	L3MBTL2	Patel (2019)	30924900
	LAMC3	Patel (2019)	30924900

	Gene	Reference	PMID
	LRRC40	Raghavan (2018)	30009200
	LRRK2	Zimprich (2004)	15541309
	LRRN4CL	Raghavan (2018)	30009200
	LTBP4	Raghavan (2018)	30009200
	LYG1	Raghavan (2018)	30009200
**M**	MAGI2	Raghavan (2018)	30009200
	MAMDC4	Raghavan (2018)	30009200
	MAOB	Leko (2020)	31771069
	MAPK8IP3	Raghavan (2018)	30009200
	MAPT	Patel (2019)	30924900
	MATR3	Cirulli (2015)	25700176
	MC1R	Tell-Marti (2017)	28059796
	MCM8	Raghavan (2018)	30009200
	MED16	Raghavan (2018)	30009200
	MEF2C	Lambert (2013)	24162737
	MIR3654	Vardarajan (2018)	29688227
	MMP20	Raghavan (2018)	30009200
	MMP21	Raghavan (2018)	30009200
	MPEG1	Raghavan (2018)	30009200
	MROH2B	Raghavan (2018)	30009200
	MROH9	Raghavan (2018)	30009200
	MRPL10	Raghavan (2018)	30009200
	MRPL46	Raghavan (2018)	30009200
	MRPS7	Raghavan (2018)	30009200
	MS4A	Ma (2015)	24981432
	MS4A4A	Hollingworth (2011)	21460840
	MS4A6A	Patel (2019)	30924900
	MS4A6E	Naj (2011)	21460841
	MSH3	Raghavan (2018)	30009200
	MSI2	Couthouis (2011)	22065782
	MTHFD1	Patel (2019)	30924900
	MTMR11	Raghavan (2018)	30009200
	MTO1	Raghavan (2018)	30009200
	MUC17	Zhang (2019)	30503768
	MYH9	Raghavan (2018)	30009200
	MYO1C	Raghavan (2018)	30009200
	MYO3B	Raghavan (2018)	30009200
	MYO5C	Raghavan (2018)	30009200
	MYO7B	Raghavan (2018)	30009200
	MYRF	Vardarajan (2018)	29688227
	MYT1L	Raghavan (2018)	30009200
**N**	NANS	Beecham (2018)	30569016
	NAT2	Raghavan (2018)	30009200
	NDUFB7	Raghavan (2018)	30009200
	NEFH	Cirulli (2015)	25700176
	NEK1	Cirulli (2015)	25700176
	NFX1	Raghavan (2018)	30009200
	NID2	Raghavan (2018)	30009200
	NLN	Raghavan (2018)	30009200
	NME8	Lambert (2013)	24162737
	NOL8	Raghavan (2018)	30009200
	NOSIP	Raghavan (2018)	30009200
	NOTCH3	Raghavan (2018)	30009200
	NPC1	Zhang (2019)	30503768
	NQO1	Raghavan (2018)	30009200
	NRXN3	Raghavan (2018)	30009200
	NUAK2	Raghavan (2018)	30009200
	NUDT12	Raghavan (2018)	30009200
	NUDT8	Raghavan (2018)	30009200
**O**	OAZ3	Raghavan (2018)	30009200
	OBSCN	Patel (2019)	30924900
	OGDH	Raghavan (2018)	30009200
	OPTN	Cirulli (2015)	25700176
	OR10G8	Raghavan (2018)	30009200
	OR13C4	Raghavan (2018)	30009200
	OR2K2	Raghavan (2018)	30009200
	OR2M7	Raghavan (2018)	30009200
	OR4S1	Raghavan (2018)	30009200
	OR51I2	Patel (2019)	30924900
	OR52N1	Raghavan (2018)	30009200
	OR56B1	Patel (2019)	30924900
	OR8G5	Ma (2019)	31180460
	OXNAD1	Raghavan (2018)	30009200
**P**	P2RY13	Raghavan (2018)	30009200
	PACS2	Raghavan (2018)	30009200
	PAQR8	Raghavan (2018)	30009200
	PAX2	Raghavan (2018)	30009200
	PCDHGA5	Raghavan (2018)	30009200
	PCK2	Raghavan (2018)	30009200
	PCSK9	Raghavan (2018)	30009200
	PDCD4	Raghavan (2018)	30009200
	PDE12	Raghavan (2018)	30009200
	PDGFRL	Patel (2019)	30924900
	PEX5	Raghavan (2018)	30009200
	PEX5L	Raghavan (2018)	30009200
	PFN1	Cirulli (2015)	25700176
	PGA5	Vardarajan (2018)	29688227
	PGAP3	Raghavan (2018)	30009200
	PGLYRP4	Raghavan (2018)	30009200
	PGRN	Cirulli (2015)	25700176
	PHF11	Raghavan (2018)	30009200
	PHF23	Raghavan (2018)	30009200

	Gene	Reference	PMID
	PHGDH	Raghavan (2018)	30009200
	PHIP	Raghavan (2018)	30009200
	PICALM	Harold (2009)	19734902
	PIGU	Raghavan (2018)	30009200
	PIP5K1C	Raghavan (2018)	30009200
	PLA2G4D	Raghavan (2018)	30009200
	PLAU	Thornton-Wells (2008)	18076107
	PLCG2	Conway (2018)	30326945
	PLCL1	Beecham (2018)	30569016
	PLD3	Tan (2019)	30837833
	PLD4	Patel (2019)	30924900
	PLEKHB2	Raghavan (2018)	30009200
	PLEKHG4	Raghavan (2018)	30009200
	PLXNA4	Raghavan (2018)	30009200
	POLR3B	Raghavan (2018)	30009200
	PON1	Erlich (2006)	16319130
	PON2	Erlich (2006)	16319130
	PON3	Erlich (2006)	16319130
	POU6F1	Raghavan (2018)	30009200
	PPARGC1A	Hamilton (2007)	17440948
	PPP1R14A	Patel (2019)	30924900
	PPP1R18	Raghavan (2018)	30009200
	PPP1R32	Vardarajan (2018)	29688227
	PRAM1	Raghavan (2018)	30009200
	PREX1	Raghavan (2018)	30009200
	PRKAR1B	Wong (2014)	24722252
	PRPH	Cirulli (2015)	25700176
	PSEN1	Rogaev (1995)	7651536
	PSEN2	Sherrington (1995)	7596406
	PSIP1	Raghavan (2018)	30009200
	PSMB4	Raghavan (2018)	30009200
	PSMC3	Raghavan (2018)	30009200
	PSMD2	Raghavan (2018)	30009200
	PSTPIP1	Raghavan (2018)	30009200
	PTK2B	Lambert (2013)	24162737
	PTPN7	Raghavan (2018)	30009200
	PTPRH	Raghavan (2018)	30009200
	PTPRR	Raghavan (2018)	30009200
**Q**	QKI	Raghavan (2018)	30009200
	QPCT	Raghavan (2018)	30009200
	QRICH2	Patel (2019)	30924900
	QRSL1	Raghavan (2018)	30009200
**R**	RAB38	Ferrari (2014)	24943344
	RALYL	Couthouis (2011)	22065782
	RARS	Raghavan (2018)	30009200
	RBM12B	Couthouis (2011)	22065782
	RBM14	Couthouis (2011)	22065782
	RBM4	Couthouis (2011)	22065782
	RBM41	Couthouis (2011)	22065782
	RBM4B	Couthouis (2011)	22065782
	RBM5	Couthouis (2011)	22065782
	RBM6	Raghavan (2018)	30009200
	RBM9	Couthouis (2011)	22065782
	RBMS1	Couthouis (2011)	22065782
	RBMS2	Couthouis (2011)	22065782
	RBPMS	Couthouis (2011)	22065782
	REL	Raghavan (2018)	30009200
	REM1	Raghavan (2018)	30009200
	RIC8A	Raghavan (2018)	30009200
	RIN3	Vardarajan (2018)	29688227
	RIPK2	Raghavan (2018)	30009200
	ROD1	Couthouis (2011)	22065782
	RPL36AL	Raghavan (2018)	30009200
	RPS16	Patel (2019)	30924900
	RUSC2	Raghavan (2018)	30009200
**S**	SCAPER	Raghavan (2018)	30009200
	SCFD1	Patel (2019)	30924900
	SCN4A	Zhang (2019)	30503768
	SDCCAG8	Raghavan (2018)	30009200
	SEL1L3	Raghavan (2018)	30009200
	SEPT10	Raghavan (2018)	30009200
	SERAC1	Raghavan (2018)	30009200
	SETD1B	Raghavan (2018)	30009200
	SETX	Cirulli (2015)	25700176
	SIDT1	Raghavan (2018)	30009200
	SIGMAR1	Cirulli (2015)	25700176
	SIRPB1	Raghavan (2018)	30009200
	SKIDA1	Raghavan (2018)	30009200
	SLC15A5	Beecham (2018)	30569016
	SLC22A8	Raghavan (2018)	30009200
	SLC24A1	Raghavan (2018)	30009200
	SLC24A3	Ma (2019)	31180460
	SLC24A4	Lambert (2013)	24162737
	SLC2A5	Raghavan (2018)	30009200
	SLC3A2	Vardarajan (2018)	29688227
	SLC44A4	Raghavan (2018)	30009200
	SLC52A1	Raghavan (2018)	30009200
	SMAD3	Raghavan (2018)	30009200
	SMCR8	Raghavan (2018)	30009200
	SMPDL3A	Raghavan (2018)	30009200
	SNED1	Raghavan (2018)	30009200
	SNRPA	Couthouis (2011)	22065782

	Gene	Reference	PMID
	SNRPB2	Couthouis (2011)	22065782
	SNX8	Raghavan (2018)	30009200
	SOD1	Cirulli (2015)	25700176
	SORCS2	Patel (2019)	30924900
	SORL1	Raghavan (2018)	30009200
	SPAST	Cirulli (2015)	25700176
	SPATA6	Raghavan (2018)	30009200
	SPATA7	Patel (2019)	30924900
	SPEN	Raghavan (2018)	30009200
	SPG11	Cirulli (2015)	25700176
	SPHK2	Patel (2019)	30924900
	SPTB	Raghavan (2018)	30009200
	SQSTM1	Cirulli (2015)	25700176
	SS18L1	Cirulli (2015)	25700176
	ST5	Raghavan (2018)	30009200
	ST6GAL2	Raghavan (2018)	30009200
	STAB1	Zhang (2019)	30503768
	STARD7	Raghavan (2018)	30009200
	STK38L	Raghavan (2018)	30009200
	STT3A	Raghavan (2018)	30009200
	SUV420H1	Patel (2019)	30924900
**T**	TAF15	Couthouis (2011)	22065782
	TARBP1	Raghavan (2018)	30009200
	TARDBP	Couthouis (2011)	22065782
	TAS1R3	Patel (2019)	30924900
	TBK1	Cirulli (2015)	25700176
	TBL2	Raghavan (2018)	30009200
	TCOF1	Raghavan (2018)	30009200
	TET3	Raghavan (2018)	32330418
	TFDP2	Raghavan (2018)	30009200
	THNSL1	Patel (2019)	30009200
	TIA1	Couthouis (2011)	30924900
	TM2D3	Patel (2019)	22065782
	TMEM106B	Van Deerlin (2010)	30924900
	TMEM132A	Vardarajan (2018)	20154673
	TMEM134	Raghavan (2018)	29688227
	TMEM232	Raghavan (2018)	30009200
	TMEM253	Raghavan (2018)	30009200
	TMEM38A	Raghavan (2018)	30009200
	TMIGD2	Raghavan (2018)	30009200
	TP53	Raghavan (2018)	30009200
	TPRG1L	Raghavan (2018)	30009200
	TREM2	Patel (2019)	30009200
	TRIM56	Raghavan (2018)	30924900
	TRMT1	Raghavan (2018)	30009200
	TRPC4AP	Poduslo (2009)	18449908
	TRPV2	Raghavan (2018)	30009200
	TSTD2	Raghavan (2018)	30009200
	TTC3	Beecham (2018)	30569016
	TTC36	Raghavan (2018)	30009200
	TTN	Raghavan (2018)	30009200
	TUBA4A	Cirulli (2015)	25700176
	TULP1	Raghavan (2018)	30009200
	TVP23B	Raghavan (2018)	30009200
**U**	UBAP2	Patel (2019)	30924900
	UBE2O	Raghavan (2018)	30009200
	UBQLN2	Cirulli (2015)	25700176
	UBR4	Raghavan (2018)	30009200
	UBXN2A	Raghavan (2018)	30009200
	UFL1	Raghavan (2018)	30009200
	ULK2	Raghavan (2018)	30009200
	ULK4	Raghavan (2018)	30009200
	UNC13A	Karch (2018)	29630712
	UNC5C	Wetzel-Smith (2014)	29951500
	USP32	Patel (2019)	30924900
**V**	VAPB	Cirulli (2015)	25700176
	VCP	Cirulli (2015)	25700176
	VEGF	Del Bo (2005)	15732116
	VIPR1	Raghavan (2018)	30009200
	VKORC1	Marioni (2018)	29777097
	VPS13C	Lesage (2016)	26942284
**W**	WDR11	Raghavan (2018)	30009200
	WDR73	Raghavan (2018)	30009200
	WDR76	Raghavan (2018)	30009200
	WDR87	Raghavan (2018)	30009200
	WDTC1	Raghavan (2018)	30009200
**X**	XRCC1	Raghavan (2018)	30009200
	XRCC3	Raghavan (2018)	30009200
**Z**	ZBTB4	Raghavan (2018)	30009200
	ZC3HAV1	Raghavan (2018)	30009200
	ZCWPW1	Raghavan (2018)	30009200
	ZDHHC3	Raghavan (2018)	30009200
	ZHX2	Raghavan (2018)	30009200
	ZNF135	Raghavan (2018)	30009200
	ZNF169	Raghavan (2018)	30009200
	ZNF180	Raghavan (2018)	30009200
	ZNF333	Zhang (2019)	30503768
	ZNF366	Raghavan (2018)	30009200
	ZNF467	Raghavan (2018)	30009200
	ZNF528	Raghavan (2018)	30009200
	ZNF571	Raghavan (2018)	30009200
	ZNF576	Raghavan (2018)	30009200

Gene	Reference	PMID
ZNF610	Raghavan (2018)	30009200
ZNF611	Raghavan (2018)	30009200
ZNF665	Raghavan (2018)	30009200
ZNF683	Raghavan (2018)	30009200
ZNF726	Raghavan (2018)	30009200
ZNF747	Raghavan (2018)	30009200
ZNF76	Raghavan (2018)	30009200
ZNF778	Raghavan (2018)	30009200
ZSWIM2	Raghavan (2018)	30009200

The list includes genes that have been associated with risk for dementia in previous studies. Rare variants in our cohort were compared with this list and evaluated in more detail subsequently. PMID: PubMed Unique Identifier of reference paper used for curation of gene list. Underlined genes indicate potentially pathogenic variants identified in those genes in our cohort, further described in the paper.
